# The ameliorative role of phlorotannin on aflatoxin B_1_-induced liver oxidative stress and mitochondrial injury is related to the activation of Nrf2 and Nrf1 signaling pathways in broilers

**DOI:** 10.1186/s40104-025-01210-z

**Published:** 2025-05-22

**Authors:** Xueqing Ye, Yuying Yang, Qinghua Yao, Mengyi Huang, Balamuralikrishnan Balasubramanian, Rajesh Jha, Wenchao Liu

**Affiliations:** 1 Department of Animal Science, College of Coastal Agricultural Sciences, Guangdong Ocean University, Zhanjiang, 524088 China; 2 Department of Food Science and Biotechnology, College of Life Science, Sejong University, Seoul, 05006 South Korea; 3 Department of Human Nutrition, Food and Animal Sciences, College of Tropical Agriculture and Human Resilience, University of Hawaii at Manoa, Honolulu, HI 96822 USA

**Keywords:** Aflatoxin B_1_, Biological detoxification, Broiler chickens, Liver injury, Phlorotannin

## Abstract

**Background:**

Aflatoxin B_1_ (AFB_1_) risks animal and human health, and the liver is considered the most crucial detoxification organ. Phlorotannin (PT) is a polyhydroxy phenol that has a wide range of biological activities, including anti-oxidation and hepatoprotection, which can promote the ability of liver detoxification. This study aimed to elucidate the protective effect of PT on AFB_1_-induced liver damage in broilers.

**Results:**

In vivo experiment showed that the PT reduced AFB_1_ content and AFB_1_-exo-8,9-epoxide DNA (AFBO-DNA) concentration in serum and liver (*P* < 0.05), improved the histomorphology of liver and hepatic mitochondria, and activated nuclear factor erythroid 2-related factor 2 (Nrf2)-related antioxidant and detoxification pathway by upregulating the activities of antioxidant enzymes (catalase [CAT], glutathione S-transferase [GST]) and total antioxidant capacity (T-AOC) level (*P* < 0.05), and inhibited the mRNA expression of *CYP1A1 *(cytochrome P450 family 1 subfamily A member 1) and phase II detoxification enzyme related genes (*GPX1*, *GSTT1*, and *NQO1*) of broilers exposed to AFB_1 _(*P* < 0.05). Meanwhile, PT upregulated the Nrf1 pathway-related mitochondrial biosynthetic genes (*Nrf1*, mitochondrial transcription factor A [*TFAM*], mitofusin 1 [*MFN1*]) in broilers fed AFB_1_ contaminated diet (*P* < 0.05). In vitro verification study suggested that the use of Nrf2/Nrf1 inhibitors suppressed the ameliorative role of PT on AFB_1_-induced liver injury of broilers, which was manifested in the mRNA expression of *Nrf2*, *NQO1*, *GSTT3*, *Nrf1*, *TFAM*, and other genes decreasing (*P* < 0.05), and down-regulation of the protein expression of Nrf2, total and nucleus p-Nrf2, and total and nucleus p-Nrf1 (*P* < 0.05).

**Conclusion:**

The PT ameliorates oxidative stress and hepatotoxicity by activating the Nrf2-mediated phase II detoxification enzymes pathway and maintains mitochondrial homeostasis by activating the Nrf1 signaling pathway in broilers exposed to AFB_1_.

**Graphical Abstract:**

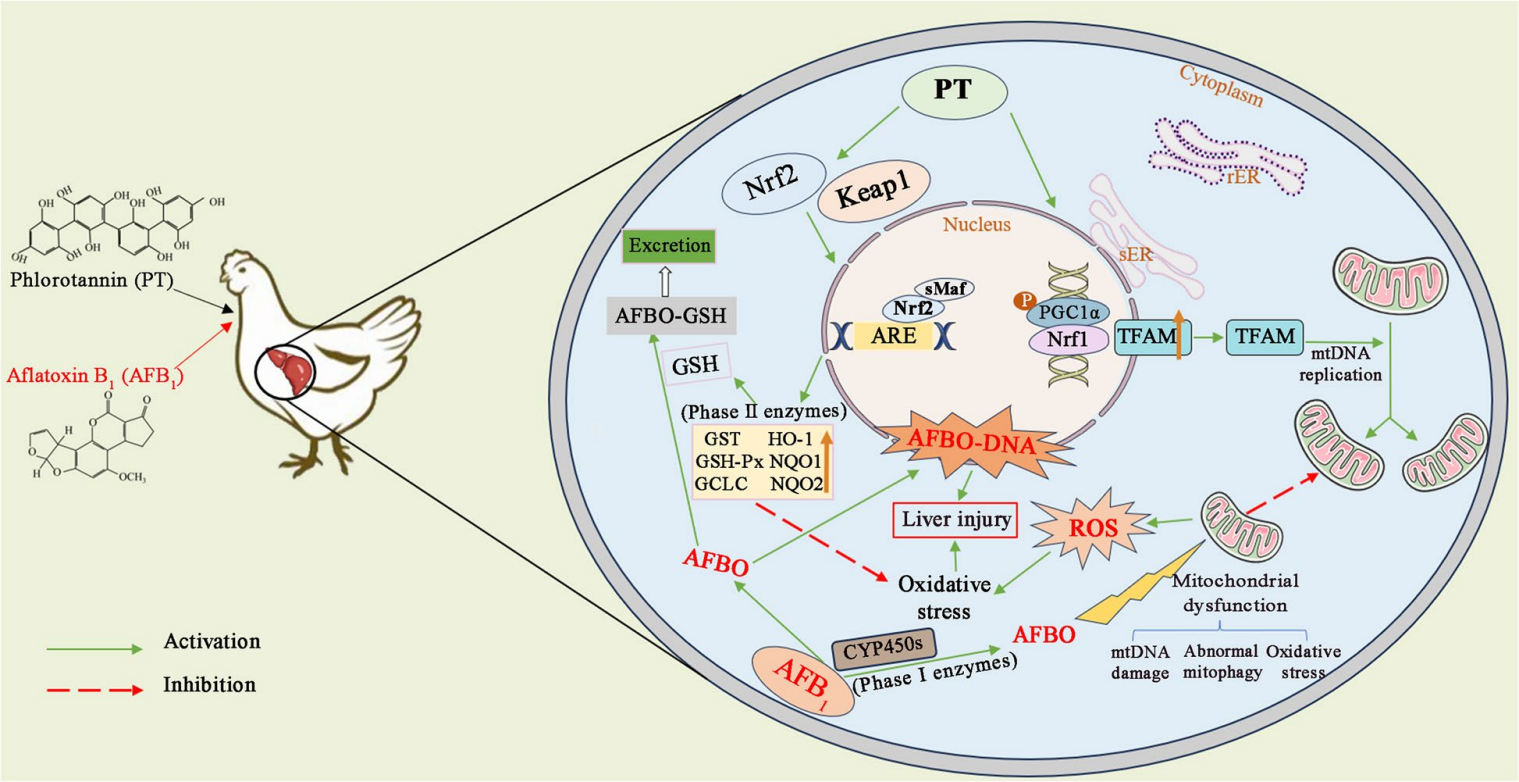

**Supplementary Information:**

The online version contains supplementary material available at 10.1186/s40104-025-01210-z.

## Introduction

Mycotoxin contamination in foodstuffs and feed ingredients causes substantial economic losses in the food and agricultural industry, and the accumulation of mycotoxin has significant toxic effects on humans and animals [1]. Among these, aflatoxin B_1_ (AFB_1_) is one of the most prevalent; it is a secondary metabolite produced by *Aspergillus flavus* and *Aspergillus parasiticus* [2, 3]. According to the global data reported in the past ten years, the incidence and the highest level of aflatoxin in cereals are 55% and 1,642 μg/kg, respectively [4]. Feeding AFB_1_-contained diets negatively impacts livestock production and causes a significant threat to food security [5, 6]. Chicken is currently the main animal-derived food worldwide. It has been reported that broiler chickens are sensitive to AFB_1_, and AFB_1_ induces oxidative stress and hepatotoxicity in broilers, which is detrimental to broiler production and supply [7, 8].

The AFB_1_ is classified as a carcinogen because it produces harmful product AFB_1_-exo-8,9-epoxide (AFBO) through the biotransformation pathway of metabolism and detoxification. AFBO can bind to DNA and proteins in cells to form adducts, resulting in DNA mutations and liver damage [9, 10]. It has been found that when the body is exposed to AFB_1_, it will not only lead to the oxidative phosphorylation decoupling of the mitochondrial respiratory chain and disturb the oxidative balance of hepatocytes but also reduce the mitochondrial membrane potential, cause mitochondrial swelling and induce mitochondrial dysfunction [11, 12]. It has been proved that the activation of AFB_1_ into AFBO is regulated by cytochrome P (CYP) enzymes (phase I metabolic enzymes), which mainly include CYP1A1, CYP1A2, CYPA6, CYP3A4 [13]. Subsequently, AFBO is detoxified by conjugation to reduced glutathione (GSH), which is catalyzed by glutathione S-transferase (GST; phase II detoxification enzyme) [14]. As is known, the liver is the main organ for the metabolism and detoxification of AFB_1_ [15]. When the content of AFB_1_ exceeds the detoxification threshold of the liver, excessive AFB_1_ produces a large amount of reactive oxygen species (ROS) during the metabolic process in the liver, thus causing oxidative stress and impairing the hepatic function [16, 17]. The nuclear transcription factor is closely related to the process of cell oxidation, in which nuclear factor erythroid 2-related factor 2 (Nrf2) plays an irreplaceable role in regulating the process of oxidative stress [18]. On the other hand, mitochondria are important organelles in the hepatocytes and are involved in producing energy and ROS [19, 20]. It has also been reported that the metabolism and detoxification of AFB_1_ are closely related to mitochondria [21, 22]. Several transcription factors can regulate the biogenesis and function of mitochondria, such as peroxisome proliferators-activated receptor γ coactivator 1 alpha (PGC-1α), nuclear respiratory factor 1 (Nrf1), and mitochondrial transcription factor A (TFAM) [23]. Exposure to AFB_1_ impairs the structure of mitochondria, leading to the disorder of its biosynthesis function and further accelerating liver damage [24].

Many studies have shown that several bioactive substances can alleviate AFB_1_-induced liver injury by reducing oxidative stress and mitochondrial-mediated apoptosis [25–27]. Polyphenols are natural antioxidants because they contain one or more hydroxyl groups in the structures and have antioxidant, anti-tumor, and anti-inflammatory functions [28, 29]. Polyphenols are abundant in plants and have been reported to relieve AFB_1_-induced hepatotoxicity, nephrotoxicity, reproductive toxicity, inflammatory reaction, and apoptosis in vitro and in vivo by reducing oxidative stress [15, 30]. With the continuous development of marine resources, the development and utilization of seaweed resources have made remarkable progress in recent years. As a secondary metabolite of brown algae, phlorotannin (PT) is a polyhydroxy phenol that has a wide range of biological activities [31], including anti-oxidation [32], anti-inflammation, and hepatoprotection [33]. A previous study confirmed that PT plays a preventive role in liver injury induced by toxins [34]. However, it remains unclear whether PT exerts a beneficial role in AFB_1_-induced liver injury in broilers, especially the mitochondrial protective effect of PT in the liver is still unknown. Therefore, this study was conducted to evaluate the effects of PT on the liver injury of broilers exposed to AFB_1_ and to illustrate the molecular mechanisms to provide new strategies for alleviating the hepatotoxicity of AFB_1_ in broilers.

## Materials and methods

### Experimental birds and diets (in vivo)

All experimental procedures were conducted with the approval of the Animal Care and Use Committee of College of Coastal Agricultural Sciences of Guangdong Ocean University (Approval No. 20221008, Zhanjiang, Guangdong, China). The PT was obtained from Shaanxi Baichuan Biotechnology Co., Ltd. (Xi’an, China). The AFB_1_ was purchased from Sigma-Aldrich (St. Louis, MO, USA). A total of 360 one-day-old Abor Acres (AA) male broilers (Charoen Pokphand Group, Zhanjiang, China) were randomly and equally divided into 6 groups: Control (basal diet), AFB_1_ (0.1 mg/kg AFB_1_), AFB_1_ + PT_200_ (0.1 mg/kg AFB_1_ + 200 mg/kg PT), AFB_1_ + PT_400_ (0.1 mg/kg AFB_1_ + 400 mg/kg PT), AFB_1_ + PT_600_ (0.1 mg/kg AFB_1_ + 600 mg/kg PT), AFB_1_ + PT_800_ (0.1 mg/kg AFB_1_ + 800 mg/kg PT). There were 6 replicates and 10 birds per replicate. Birds of each replication were housed in wire cages (80 cm length × 70 cm width × 40 cm height) equipped with nipple waterer, for a feeding period of 21 d. The doses and the experimental cycle were chosen on the basis of previous studies, which reported that dietary consumption of 0.1 mg/kg AFB_1_ for 21 d induced liver damage in broiler chickens [35, 36]. The temperature remained around 33–35 °C for the first three days, then reduced gradually (2–3 °C every week) from 35 to 26 °C, and the relative humidity was maintained at 60%–70%. The basal diets were formulated to meet the nutrient requirements of broilers (NRC, 1994) [37] and feeding standards for the AA [38]. AFB_1_ is dissolved in methanol to make a solution, and then evenly sprayed into the basal diet and mixed to obtain the 0.1 mg/kg AFB_1_-contaminated diet [39]. The equivalent methanol was sprayed evenly on the normal feed to obtain the basal diet. The treatment concentration of PT was calculated and added uniformly into the diet and mixed evenly. All the diets were evaporated at 37 °C and mixed with a vertical mixer. The ingredient composition and nutritional levels of the basal diet are shown in Table 1.

**Table 1:** Compositions and nutrient levels of experimental basal diet (as-fed basis)

Ingredients	Contents, %	Nutrient levels	Contents, %
Corn	53.00	ME, MJ/kg	12.42
Soybean meal	33.00	Crude protein	20.87
Wheat bran	4.60	Ca	1.00
Fishmeal	2.00	Total P	0.68
Soybean oil	3.00	Available P	0.46
Limestone	1.50	Lys	1.22
CaHPO_4_	1.60	Met	0.53
L-Lysine	0.10	Total Met + Cys	0.87
DL-Methionine	0.20		
NaCl	0.30		
Choline chloride	0.20		
Mineral premix^1^	0.30		
Vitamin premix^2^	0.20		

^1^Premix provided per kilogram of diet: 8 mg of Cu (CuSO_4_), 80 mg of Fe (FeSO_4_), 85 mg of Mn (MnSO_4_), 80 mg of Zn (ZnSO_4_), 0.2 mg of Se (Na_2_O_3_Se), 0.15 mg of I [Ca(IO_3_)_2_]

^2^Premix provided per kilogram of diet: 9,000 IU of vitamin A, 3,240 IU of vitamin D_3_, 6 IU of vitamin E, 0.75 mg of vitamin K_3_, 1.5 mg of vitamin B_1_, 4.5 mg of vitamin B_2_, 10.5 mg of vitamin B_3_, 1.5 mg of vitamin B_6_, 0.45 mg of folic acid, and 9 mg of pantothenic acid

### Sample collection

At the end of 21 d, one bird was randomly selected from each replicate (*n* = 6/treatment). The blood samples were collected into anticoagulation tubes from broiler’s wing vein. Then, the birds were sacrificed and quickly dissected and liver samples were collected. The individual serum sample was separated by centrifuging at 3,000 r/min for 15 min and then stored at −80 °C until further analysis. The liver samples were placed in an enzyme-free tube with 4% formaldehyde solution and stored at room temperature to prepare tissue sections. In order to be used for the subsequent determination of liver function, metabolite content, antioxidant, and gene expression, the remaining samples were placed in a sterile frozen tube and quickly frozen with liquid nitrogen for storage at −80 °C until further analysis.

### Growth performance analysis

During the feeding trial, broilers were weighed, and feed consumption was recorded weekly to calculate the average daily gain (ADG), average daily feed intake (ADFI), and the feed conversion ratio (FCR). In addition, the organ weight of the liver is taken after slaughter to calculate the liver index. The formula is as follows:

$$\mathrm{Liver}\;\mathrm{index}\;(\%)=\left[\mathrm{liver}\;\mathrm{weight}\;\left(\mathrm g\right)/\mathrm{body}\;\mathrm{weight}\;\left(\mathrm g\right)\right]\times100\%$$

### H&E staining

Liver tissue samples were fixed in 4% paraformaldehyde and stained with hematoxylin and eosin (H&E). The slices were placed under a 200 × microscope, and 20 visual fields were evaluated with a score of 0 for no lesions, 1 point for less than 3 lesions, 2 points for more than 3 and less than 5 lesions, and 3 points for more than 5 lesions. Images were observed with a fluorescence microscope (GD-30REL) under 200 × and 400 × magnification and captured by CapStudio software (Version No. 3.8.6).

### Terminal deoxynucleotidyl transferase-mediated dUTP nick-end labeling (TUNEL) assay

The single apoptotic nucleus or apoptotic body was stained in situ by fluorescence TUNEL assay. First, paraffin sections were dewaxed to water, and then protease K working solution (original solution:PBS = 1:9) was dripped to cover the tissues. After a series of steps, such as breaking the membrane, balancing at room temperature, adding TUNEL reaction solution, DAPI counterstaining the nucleus, and sealing the slices, the sections were viewed under a fluorescence microscope (GD-30REL), and the apoptosis rate was calculated. Principally, the nucleus stained by DAPI is blue under the excitation of ultraviolet light, the TUNEL kit (Wuhan Sevier Biotechnology Co., Ltd., Wuhan, China) is labeled with TMR fluorescein, and the positive apoptotic nucleus is red.

### Transmission electron microscope (TEM)

The fresh liver tissues were cut into 2 mm^3^ portions and fixed overnight at 4 °C with 2.5% glutaraldehyde. Then, washed three times with PBS for 15 min, and 1% osmium tetroxide at 4 °C for 2 h to fix. The slices were made by dehydration, embedding, sectioning, and staining. Finally, the mitochondrial structures were observed and recording using a TEM.

### Hepatic function, AFB_1_ metabolite content, and antioxidant indexes

The activities of aspartate aminotransferase (AST) and alanine aminotransferase (ALT) and the concentrations of total protein (TP) and albumin (ALB) were determined using the corresponding kits from the Nanjing Jiancheng Institute of Biological Engineering (Nanjing, China). The contents of AFB_1_ residues and AFBO-DNA adducts in the serum and liver of broilers were detected by ELISA kits produced by Jiangsu Meimian Industrial Co., Ltd. (Nanjing, China). The content of malondialdehyde (MDA), the activities of catalase (CAT), glutathione peroxidase (GSH-Px), glutathione S-transferase (GSH-ST), total superoxide dismutase (T-SOD), and the level of total antioxidant capacity (T-AOC) in the liver were measured using the commercial kits provided by Nanjing Jiancheng Bioengineering Research Institute Co., Ltd. (Nanjing, China). The heme oxygenase 1 (HO-1) and cytochrome P450 (CYP450) were determined by ELISA kits from the Jiangsu Meimian Industrial Co., Ltd. (Nanjing, China) according to the manufacturer's instructions.

### Quantitative real-time PCR analysis

The total RNA was extracted from about 0.1 g liver sample using 1 mL RNA lysate (TRIzol reagent) following the manufacturer’s instructions and transcribed into cDNA using a reverse transcription kit from the Novizan Co., Ltd. (Nanjing, China). RT-qPCR was conducted using qPCR SYBR Green Master Mix kit from Novizan Co., Ltd. (Nanjing, China) and Bio RAD CFX Connect PCR instrument (Hercules, CA, USA). The qPCR reaction system was as follows: Mix (10 μL), H_2_O (8.2 μL), cDNA (1 μL), F/R (0.4/0.4 μL). The PCR procedure was as follows: 95 °C for 30 s, and then 40 cycles using a step program (95 °C for 5 s and 60 °C for 34 s), followed by 1 cycle of 95 °C for 15 s, 60 °C for 1 min, and 95 °C for 15 s. The expression of the target genes was determined using the 2^−△△CT^ method, and the mRNA level of the housekeeping gene β-actin was used as an endogenous reference control. The primer sequence information is listed in Table S1.

### Leghorn Male Hepatoma cell line culture

The Leghorn Male Hepatoma (LMH) cell line was obtained from iCell Bioscience, Inc. (Shanghai, China). The LMH cells were grown on 0.1% gelatin-coated culture dishes in DMEM/F12 (Gibco, New York, USA), supplemented with 10% fetal bovine serum (FBS) (SERANA, Shanghai, China), and 1% antibiotics (penicillin and streptomycin; Gibco, New York, USA) at 37 °C with 5% CO_2_.

### Study on the toxic effect of PT on LMH cells

The LMH cells were cultured in 96‐well plates until 70%–80% confluence. Cells were treated with 0.025% DMSO as control and with PT at various concentrations (1.25, 2.5, 5, 10, 20, and 40 μg/mL). After treatment, 10 μL of the cell counting Kit-8 assay solution (CCK-8) (ZETA life, Shanghai, China) was added to each well and incubated for another 2 h. Then, the optical densities were read on a microplate reader (Bio Tek, Winooski, VT, USA) at 450 nm. At last, cell viability was calculated relative to the control group.

### Study on the effect of PT on LMH cells under the action of AFB_1_

To verify the effect of different concentrations of PT on the survival rate of LMH cells induced by AFB_1_, the experiment was divided into the following 7 groups, including the control group containing 0.025% DMSO, the AFB_1_ group containing 0.1 μg/mL AFB_1_ and the AFB_1_ + PT groups were containing 0.1 μg/mL AFB_1_ + PT (0, 1, 2, 3, 4, 5 μg/mL). According to previous studies, treating with 0.1 μg/mL AFB_1_ to the medium and incubating for 12 h had some damage to LMH cells [35]. The detection method was the same as those described in “Study on the toxic effect of PT on LMH cells”.

### In vitro verification of PT’s action pathway on AFB_1_-induced liver damage

To verify whether PT alleviates AFB_1_-induced injury of LMH cells through the Nrf2 or Nrf1 pathway, the cells were divided into 4 groups: control group (containing 0.025% DMSO), AFB_1_ group (0.1 μg/mL AFB_1_), 0.1 μg/mL AFB_1_ + 1 μg/mL PT group and 0.1 μg/mL AFB_1_ + 1 μg/mL PT + 1.9 μmol/L ML385 group or 0.1 μg/mL AFB_1_ + 1 μg/mL PT + 10 μmol/L WRR139 group. ML385 is an Nrf2 inhibitor and WRR139 is an Nrf1 inhibitor; both were purchased from MedChemExpress (Shanghai, China). The well-grown LMH cells were seeded in 6-well plates at 70%–80% confluence, and the cells were treated for 12 h. Then, the experimental steps of qPCR were the same as described above.

### Western blot analysis of protein expression in vitro

The cells were lysed with RIPA buffer (containing protease and phosphatase inhibitors; Servicebio, Wuhan, China). SDS-PAGE gels were used to electrophorese the protein samples, which were then transferred to PVDF membranes (Millipore, Burlington, MA, USA). The membranes were blocked with 5% non-fat milk in Tris-buffered-saline with Tween (TBST) for 2 h at room temperature, then incubated with primary antibodies overnight at 4 °C. After three washes with TBST, the membranes were incubated with a secondary antibody for 2 h at room temperature. Finally, the membranes were washed with TBST three times, and a Tanon 4600 System (Shanghai, China) was used to detect the expression of proteins with an enhanced chemiluminescence (ECL) kit (Tanon, Shanghai, China). The protein bands were scanned and quantified based on optical densities using ImageJ software (Version No. 1.5.4) and normalized to β-actin (Total protein) or Lamin B (Nuclear protein). Primary antibodies used were rabbit anti-Nrf2, rabbit anti-phospho-Nrf2 (ser 40), rabbit anti-Nrf1, rabbit anti-β-actin, and rabbit anti-Lamin B. Secondary antibody was used Goat Anti-Rabbit IgG (H + L). The antibody information is shown in Table S2.

### Statistical analysis

All data were analyzed by one-way ANOVA using SAS 9.4 software (Cary, NC, USA), after checking the normal distribution and homogeneity of variances, and the difference between the mean values were analyzed by Duncan’s multiple comparison. *P* < 0.05 indicates a significant difference, and 0.05 ≤ *P* < 0.10 indicates that it tends to be significant.

## Results

### PT improves growth performance and hepatic function in broilers exposed to AFB_1_

**Fig. 1:**
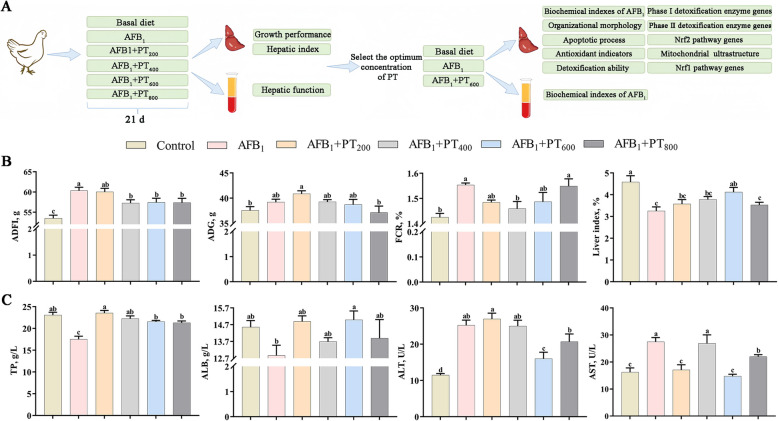
Effect of phlorotannin on AFB_1_-induced the growth performance and hepatic function in broilers. **A** Outline of experimental procedures to examine the role of PT in AFB_1_-induced broilers. **B** Growth performance and liver index. ADFI, Average daily feed intake; ADG, Average daily gain; F/G, Average daily feed intake/Average daily gain. **C** Serum TP and ALB contents and serum ALT and AST activity in the indicated broilers. TP, Total protein; ALB, Albumin; ALT, Alanine aminotransferase; AST, Aspartate transaminase. AFB_1_, Aflatoxin B_1_; PT, Phlorotannin; Control, basal diet; AFB_1_, basal diet + 0.1 mg/kg AFB_1_; AFB_1_ + PT_200_, basal diet + 0.1 mg/kg AFB_1_ + 200 mg/kg PT; AFB_1_ + PT_400_, basal diet + 0.1 mg/kg AFB_1_ + 400 mg/kg PT; AFB_1_ + PT_600_, basal diet + 0.1 mg/kg AFB_1_ + 600 mg/kg PT; AFB_1_ + PT_800_, basal diet + 0.1 mg/kg AFB_1_ + 800 mg/kg PT. Data are shown as mean ± SEM (*n* = 6). ^a–d^Different superscript letters indicate significant differences (*P* < 0.05)

Compared with the control group, the ADFI and FCR of broilers in the AFB_1_ group increased, while the liver index decreased (*P* < 0.05). Compared with AFB_1_, ADFI was decreased by adding 400, 600, and 800 mg/kg PT, and FCR was decreased by adding 400 mg/kg PT (*P* < 0.05). In addition, dietary supplementation of 600 mg/kg PT increased liver index (Fig. 1B). Hepatic function showed that the serum activities of ALT and AST of broilers in the AFB_1_ group were higher than those in the control group, while the content of TP was reduced, and the content of TP was increased by supplementation of 200, 400, 600 and 800 mg/kg PT (*P* < 0.05). Supplementation of 600 mg/kg PT increased the ALB content and decreased the activity of ALT and supplementation of 200, 600, and 800 mg/kg PT decreased the activity of AST (*P* < 0.05; Fig. 1C). Based on the above results, it can be found that 600 mg/kg PT supplementation has more obvious effects on the liver index and liver function of broilers induced by AFB_1_. Therefore, PT (600 mg/kg) was selected as the best active dose.

### PT alleviates liver histomorphology and apoptosis in broilers exposed to AFB_1_

**Fig. 2:**
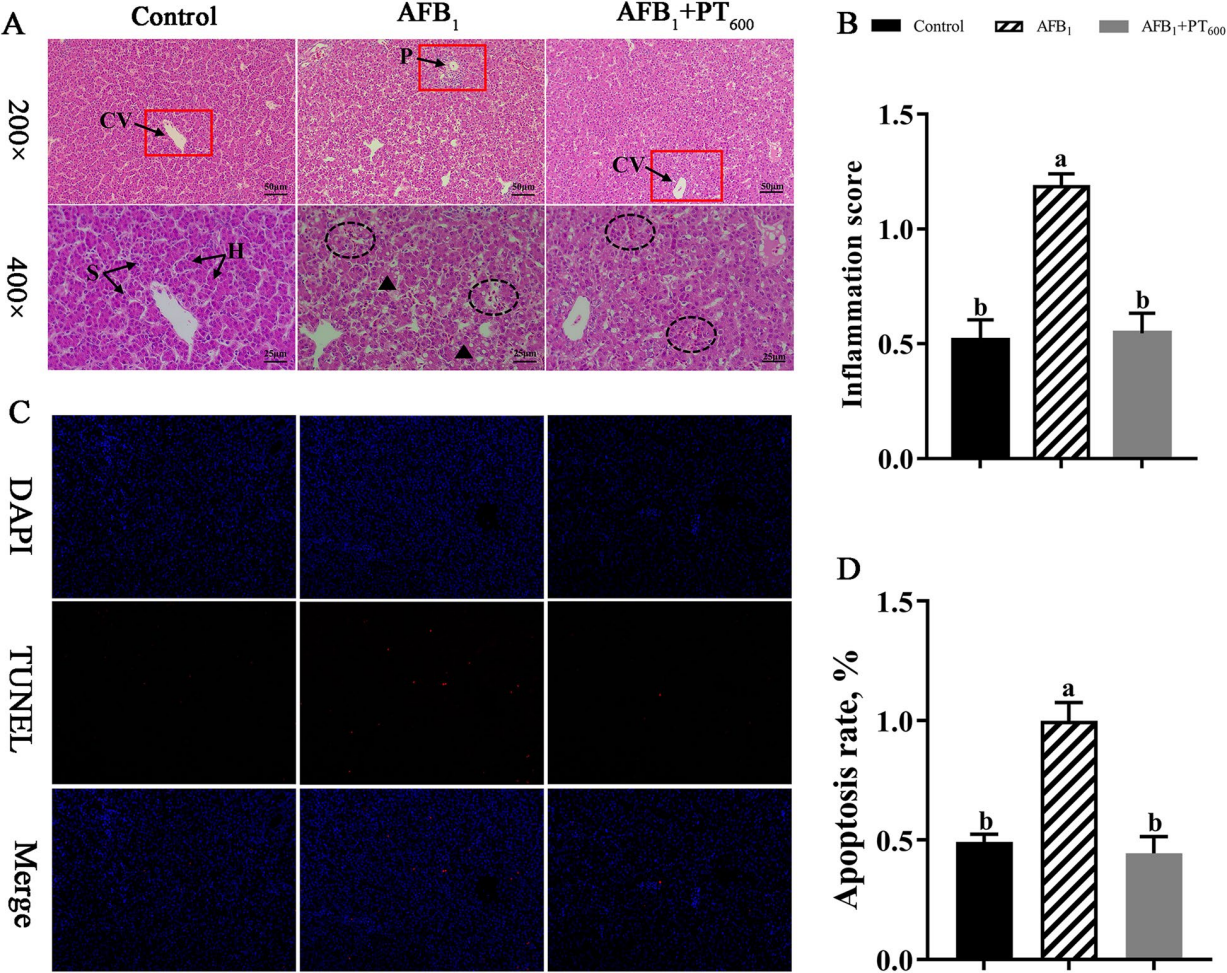
Effect of phlorotannin on AFB_1_-induced liver structure and apoptosis of broilers. **A** H&E staining images of liver sections in indicated broilers. CV: Central vein; H: Hepatocytes; S: Hepatic sinusoid; P: Portal area. Black triangle represents steatosis. Black ellipse represents inflammatory infiltration. The scar bar is 50 μm of 200 × and 25 μm of 400 ×. **B** Inflammation score by H&E staining images. **C** Representative image of immunofluorescence staining for TUNEL in broiler’s liver. The scar bar is 200 ×. **D** Apoptosis rate by TUNEL staining images. AFB_1_, Aflatoxin B_1_; PT, Phlorotannin; Control, basal diet; AFB_1_, basal diet + 0.1 mg/kg AFB_1_; AFB_1_ + PT_600_, basal diet + 0.1 mg/kg AFB_1_ + 600 mg/kg PT. Data are shown as mean ± SEM (*n* = 6). ^a,b^Different superscript letters indicate significant differences (*P* < 0.05)

The H&E staining revealed that the liver cells were arranged in a compact and normal shape and structure in the control group. Compared with the control group, the AFB_1_ group had poorly demarcated of hepatocytes, many cells showed inflammatory infiltration, some cytoplasm was loose, and there were a lot of round vacuoles with different sizes and well-demarcated, while after PT supplementation, the inflammatory infiltration of hepatocytes was reduced, the vacuoles in the cytoplasm disappeared, and the morphology and structure of hepatocytes were relieved (Fig. 2A). The scores of inflammations were higher in the AFB_1_ group than in the control group. The degree of inflammation decreased after supplementation of 600 mg/kg PT (*P* < 0.05; Fig. 2B). Using the TUNEL staining assay, it clearly observed that the apoptosis of liver cells in AFB_1_ group was more than that in the control group (Fig. 2C). The apoptosis of liver cells was decreased when PT was supplemented (*P* < 0.05; Fig. 2D).

### PT boosts hepatic antioxidant capacity and Nrf2-mediated phase II detoxification enzyme ability in broilers exposed to AFB_1_

**Fig. 3:**
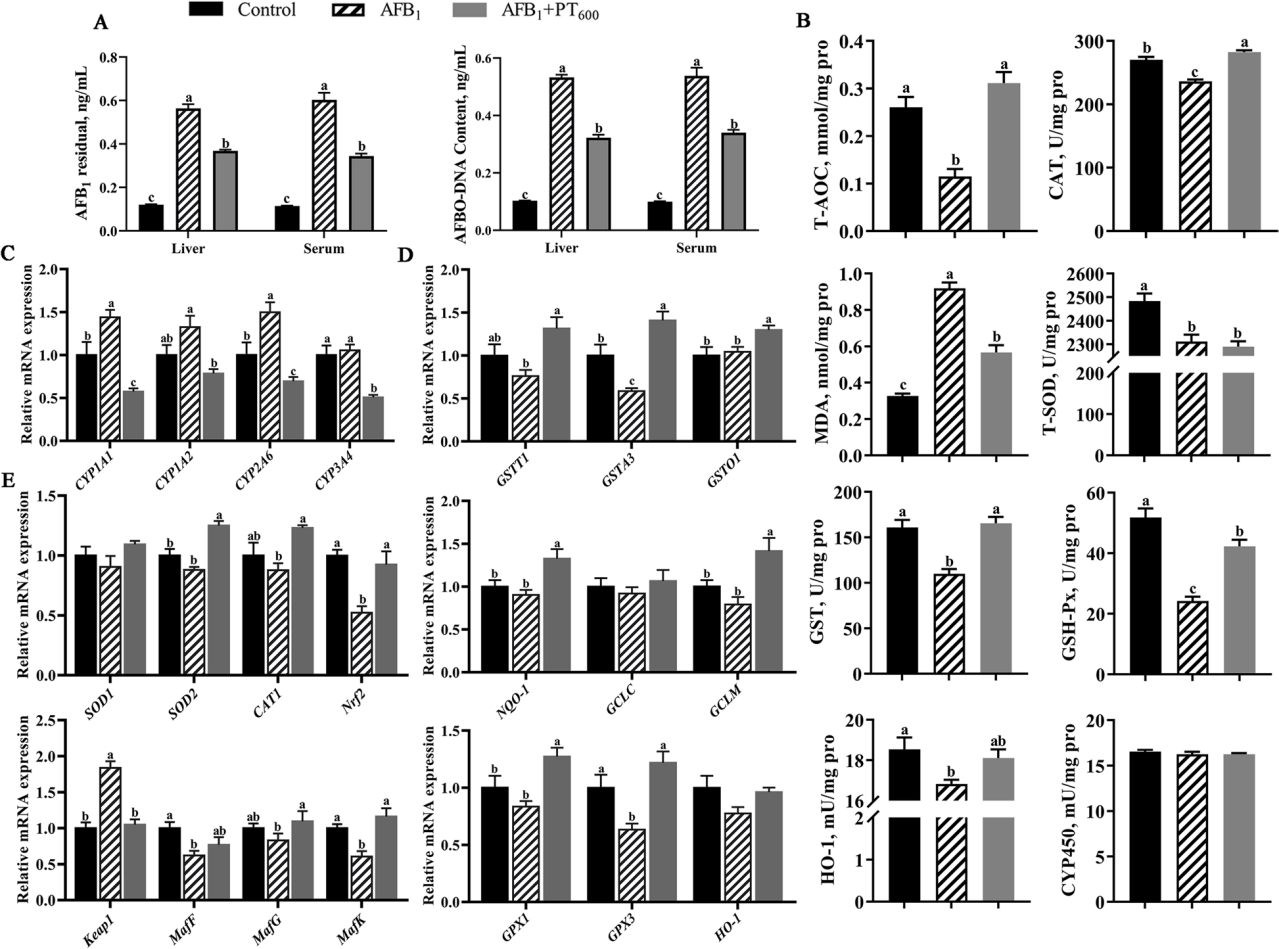
Effect of phlorotannin on AFB_1_-induced liver antioxidant capacity and Nrf2-mediated phase II etoxification enzyme ability of broilers. **A** Liver and serum AFB_1_ and AFBO contents in broilers. **B** Liver antioxidant levels and detoxification ability of broilers. **C** Relative mRNA levels of Phase I detoxification enzyme in broilers liver. **D** Relative mRNA levels of Phase II detoxification enzyme in broilers liver. **E** Relative mRNA levels of Nrf2 pathway-related gene in broilers liver. AFB_1_, Aflatoxin B_1_; PT, Phlorotannin; Control, basal diet; AFB_1_, basal diet + 0.1 mg/kg AFB_1_; AFB_1_ + PT_600_, basal diet + 0.1 mg/kg AFB_1_ + 600 mg/kg PT. Data are shown as mean ± SEM (*n* = 6). ^a–c^Different superscript letters indicate significant differences (*P* < 0.05)

The results revealed elevated AFB_1_ and AFBO-DNA contents in serum and liver of the AFB_1_ group compared with the control group, which decreased in 600 mg/kg PT treatment (*P* < 0.05; Fig. 3A). Through detecting antioxidant indexes and detoxification ability, AFB_1_ reduced T-AOC level, CAT, T-SOD, GST, GPX and HO-1 activities and increased MDA content in broiler liver compared with the control group (*P* < 0.05). Compared with the AFB_1_ group, the level of T-AOC, the activities of CAT, GST, and GPX in the liver of broilers were increased, and the content of MDA was decreased after supplementation of 600 mg/kg PT (*P* < 0.05; Fig. 3B). The q-PCR results of phase I and II detoxification enzyme related genes showed that AFB_1_ upregulated *CYP1A1* and *CYP2A6*, down-regulated *GPX3* and *GSTA3* relative expression compared with the control group (*P* < 0.05). Compared with the AFB_1_ group, PT treatment was downregulated *CYP1A1*, *CYP1A2, CYP2A6*, and *CYP3A4*, and relative expression amounts of *GPX1*,* GPX3*,* GSTT1*,* GSTA3*,* GSTO1*,* NQO1,* and Glutamate-cysteine Ligase (*GCLM)* were upregulated (*P* < 0.05; Fig. 3C and D). Nrf2 pathway-related gene expression showed that AFB_1_ decreased the relative expression of *MafF*,* MafK*, and *Nrf2* and upregulated the relative expression of *Keap1* (*P* < 0.05). In contrast, the relative expression of *MafG*,* MafK*,* SOD2*, and *Nrf2* increased, and the relative expression of *Keap1* decreased after supplementation of 600 mg/kg PT (*P* < 0.05; Fig. 3E).

### PT attenuates mitochondrial damage of the liver and upregulated the Nrf1 pathway in broilers exposed to AFB_1_

**Fig. 4:**
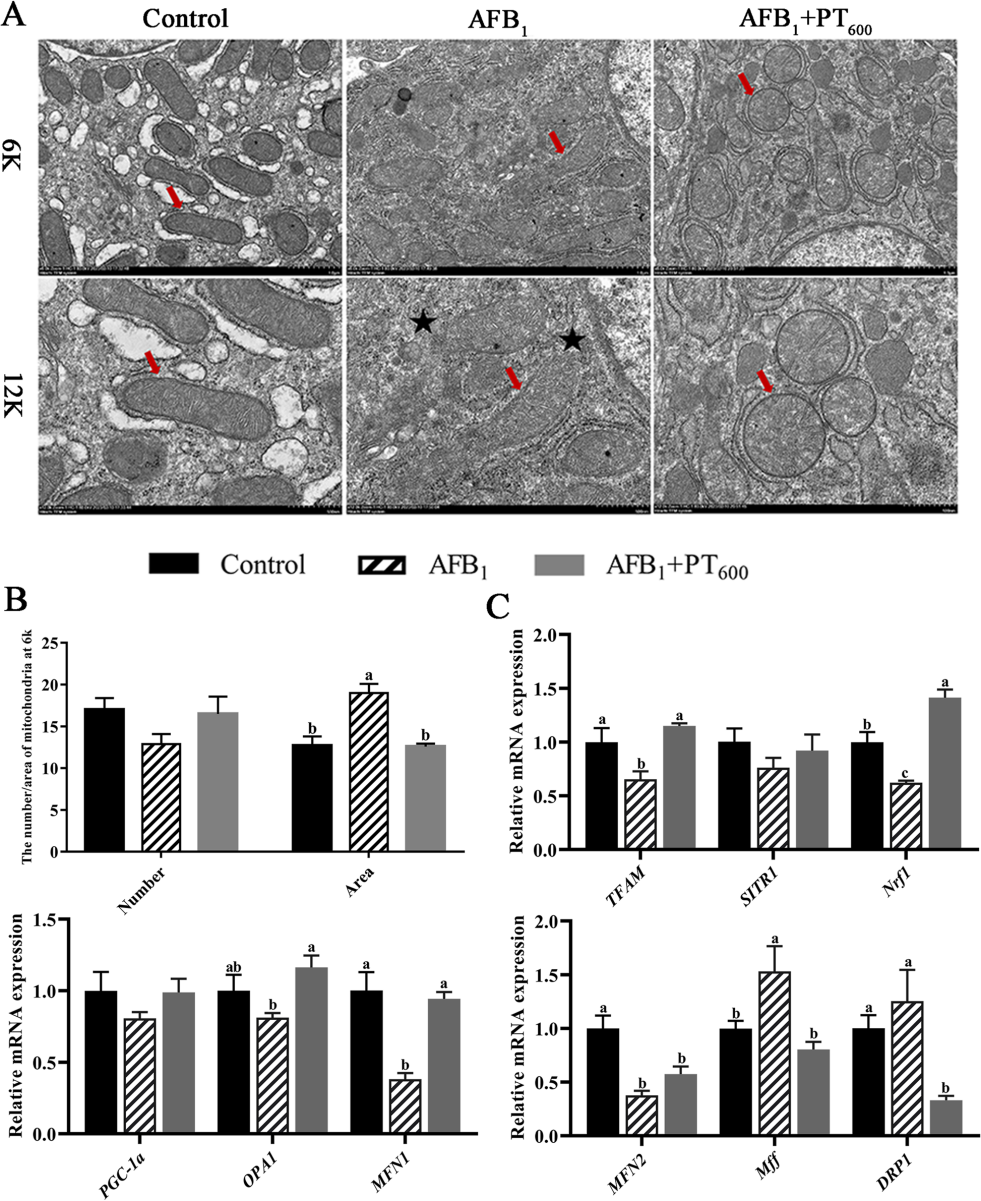
Effect of phlorotannin on AFB_1_-induced liver mitochondrial damage of broilers. **A** Transmission electron microscope image of broiler’s liver mitochondria. The scar bar is 6 K and 12 K. Red arrow represents the mitochondrial bilayer membrane structure. Black pentagram represents the blurring or disappearance of the mitochondrial bilayer membrane structure. **B** The number and area of mitochondria at 6 K. The unit of area is µm^2^. **C** Relative mRNA levels of Nrf1 pathway-related gene in broilers liver. AFB_1_, Aflatoxin B_1_; PT, Phlorotannin; Control, basal diet; AFB_1_, basal diet + 0.1 mg/kg AFB_1_; AFB_1_ + PT_600_, basal diet + 0.1 mg/kg AFB_1_ + 600 mg/kg PT. Data are shown as mean ± SEM (*n* = 6). ^a–c^Different superscript letters indicate significant differences (*P* < 0.05)

TEM images showed that the structure of mitochondria was normal, the double-layer membrane was intact and clear, and the arrangement of mitochondrial cristae was normal in the control group. In contrast, in the AFB_1_ group, the swelling of mitochondria was severe, the structure of the double-layer membrane was blurred or even disappeared, and the arrangement of Cristae was disordered. Compared with the AFB_1_ group, PT (600 mg/kg) could reduce the swelling of mitochondria and restore the structure of most mitochondrial bilayer membranes (Fig. 4A and B). Nrf1 pathway-related gene expression showed that AFB_1_ reduced the relative expression of *TFAM*, *Nrf1,* Mitofusin 1* (MFN1)*, and *MFN2* and upregulated the relative expression of *Mff* (*P* < 0.05), compared with the control group. After supplementing PT, relative expression of *TFAM, Nrf1*, optic atrophy 1 (*OPA1*), and *MFN1* were upregulated, and *Mff* and *DRP1* were down-regulated (*P* < 0.05; Fig. 4C).

### Optimal concentration of mitigative effect of PT on LMH cell injury induced by AFB_1_

**Fig. 5:**
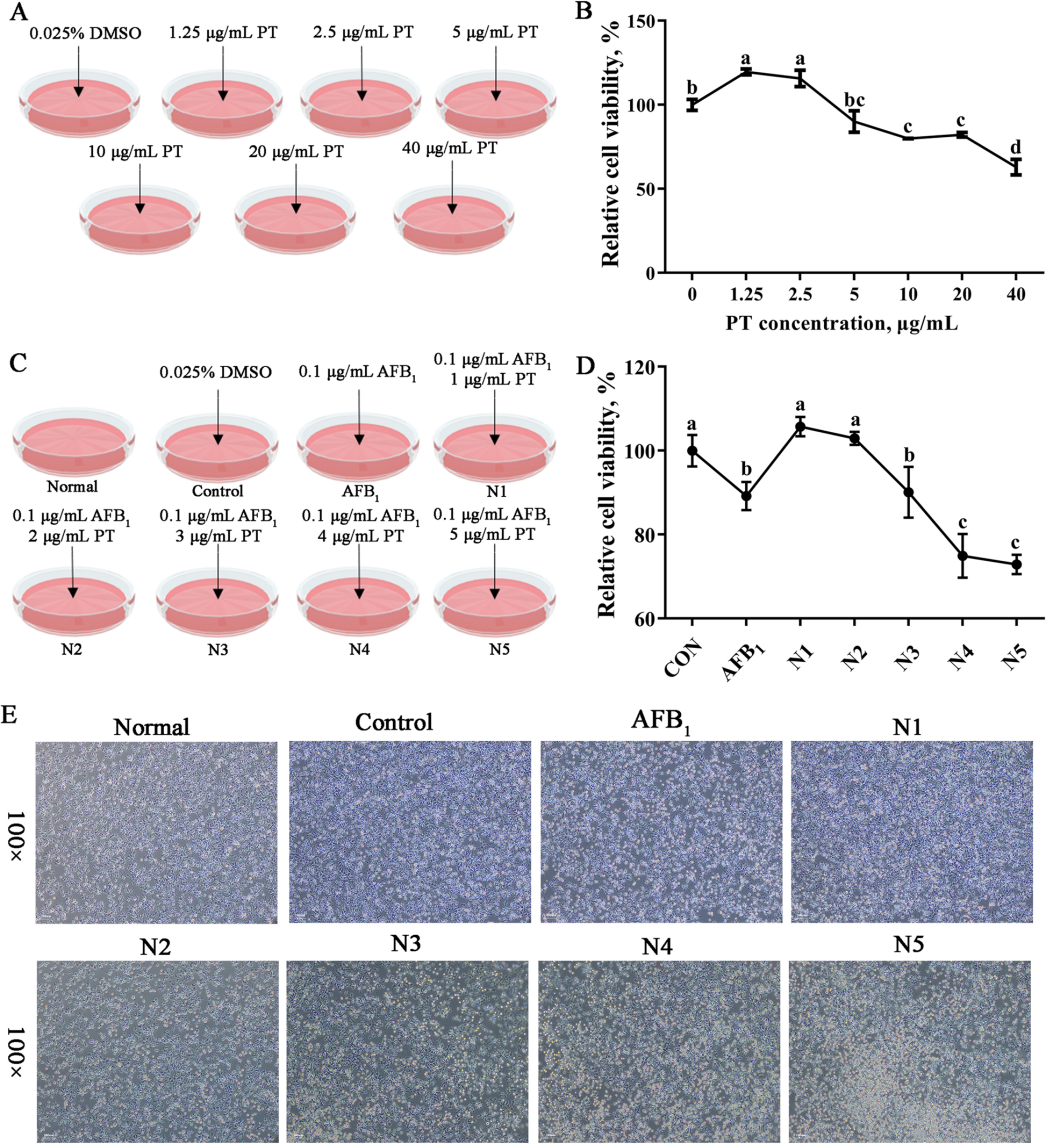
Effects of phlorotannin with different concentrations on AFB_1_-induced LMH cells. **A** Outline of the experimental grouping of different concentrations of PT on the survival rate of LMH cells. **B** Survival rate of LMH cells treated with different concentrations of PT for 12 h. **C** Outline of the experimental grouping of different concentrations of PT on the survival rate of AFB_1_-induced LMH cells. **D** Survival rate of AFB_1_-induced LMH cells treated with different concentrations of PT for 12 h. **E** LMH cell state diagram. The scar bar is 100 ×. AFB_1_, Aflatoxin B_1_; PT, Phlorotannin; Normal, 10% complete medium; Control, 10% complete medium + 0.025% DMSO; AFB_1_, 10% complete medium + 0.1 μg/mL AFB_1_; N1, 10% complete medium + 0.1 μg/mL AFB_1_ + 1 μg/mL PT; N2, 10% complete medium + 0.1 μg/mL AFB_1_ + 2 μg/mL PT; N3, 10% complete medium + 0.1 μg/mL AFB_1_ + 3 μg/mL PT; N4, 10% complete medium + 0.1 μg/mL AFB_1_ + 4 μg/mL PT; N5, 10% complete medium + 0.1 μg/mL AFB_1_ + 5 μg/mL PT. Data are shown as mean ± SEM (*n* = 6). ^a–d^Different superscript letters indicate significant differences (*P* < 0.05)

About 1.25 and 2.5 μg/mL PT increased cell survival rate compared with the control group, while concentrations of PT (10, 20, and 40 μg/mL) resulted in a survival rate declined (*P* < 0.05; Fig. 5B). Compared with the control group, AFB_1_ decreased cell survival rate, while concentrations of PT (1 and 2 μg/mL) result in increased cell survival rate (*P* < 0.05), and the cell survival rate was the highest when the concentration of PT was 1 μg/mL (Fig. 5D). Consequently, 1 μg/mL of PT was further examined for its impact on AFB_1_-induced LMH cells.

### PT reduces AFB_1_-induced oxidative stress and hepatotoxicity of LMH cells through Nrf2 pathway

**Fig. 6:**
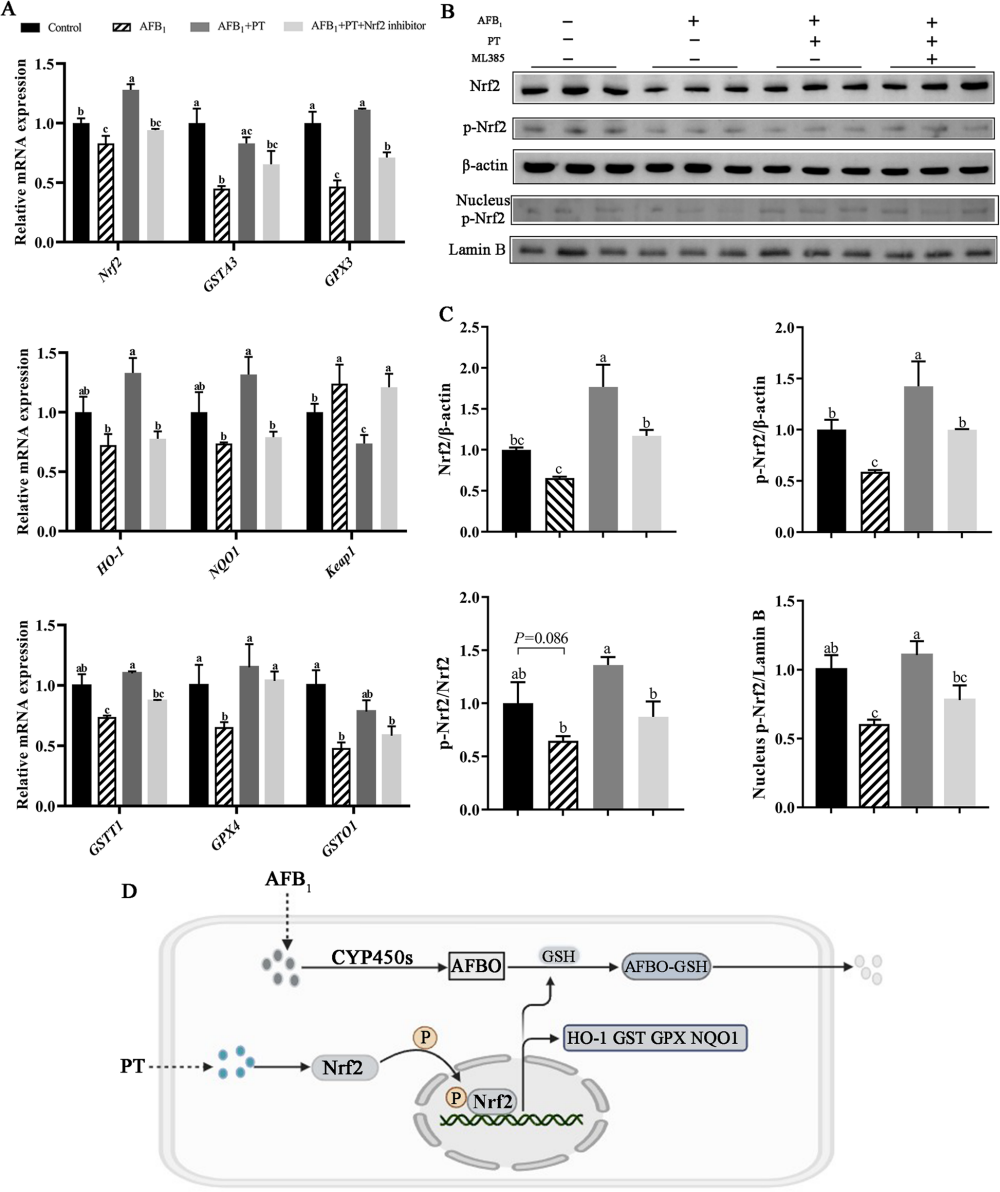
Effect of phlorotannin on AFB_1_-induced oxidative stress of LMH cells through Nrf2 pathway. **A** Relative mRNA levels of Nrf2 pathway-related gene in AFB_1_-induced LMH cells. **B** Western blot analysis of Nrf2, total and nuclear p-Nrf2 in AFB_1_-induced LMH cells. Lamin B and β-actin served as loading control. **C** Quantification of protein expression Nrf2, total and nuclear p-Nrf2. **D** Schematic diagram of AFB_1_ metabolism and PT detoxification pathway. AFB_1_, Aflatoxin B_1_; PT, Phlorotannin; Control, 10% complete medium + 0.025% DMSO; AFB_1_, 10% complete medium + 0.1 μg/mL AFB_1_; AFB_1_ + PT, 10% complete medium + 0.1 μg/mL AFB_1_ + 1 μg/mL PT; AFB_1_ + PT + Nrf2 inhibitor, 10% complete medium + 0.1 μg/mL AFB_1_ + 1 μg/mL PT + 1.9 μmol/L ML385 (Nrf2 inhibitor).– for not adding, + for adding. Data are shown as mean ± SEM (*n* = 6). ^a–c^Different superscript letters indicate significant differences (*P* < 0.05)

Compared with the control group, AFB_1_ treatment significantly downregulated the relative expression of *GSTT1*,* GPX4*,* GSTO1*,* NRF2*,* GSTA3*, and *GPX3* and upregulated the expression of *Keap1* in LMH cells (*P* < 0.05). About 1 μg/mL PT treatment promoted the expression of *GSTT1*,* GPX4*,* Nrf2*,* GSTT1*, and *GPX4* and decreased the relative expression of *Keap1* compared with the AFB_1_ group. Nrf2 inhibitor (ML385) treatment reduced the relative expression of *GSTT1, Nrf2, GPX3, HO-1*, and *NQO1* and increased the relative expression of *Keap1* (*P* < 0.05; Fig. 6A). By western blotting, compared with the control group, AFB_1_ treatment reduced the expression of p-Nrf2 protein and decreased the nuclear incorporation of p-Nrf2 (*P* < 0.05). PT treatment increased the expression of total Nrf2, p-Nrf2, and nuclear p-Nrf2 compared to the AFB_1_ group, but Nrf2 inhibitor (ML385) treatment reversed the influence compared to the PT group (*P* < 0.05; Fig. 6B and C).

### PT alleviates AFB_1_-induced mitochondrial damage of LMH cells via Nrf1 pathway

**Fig. 7:**
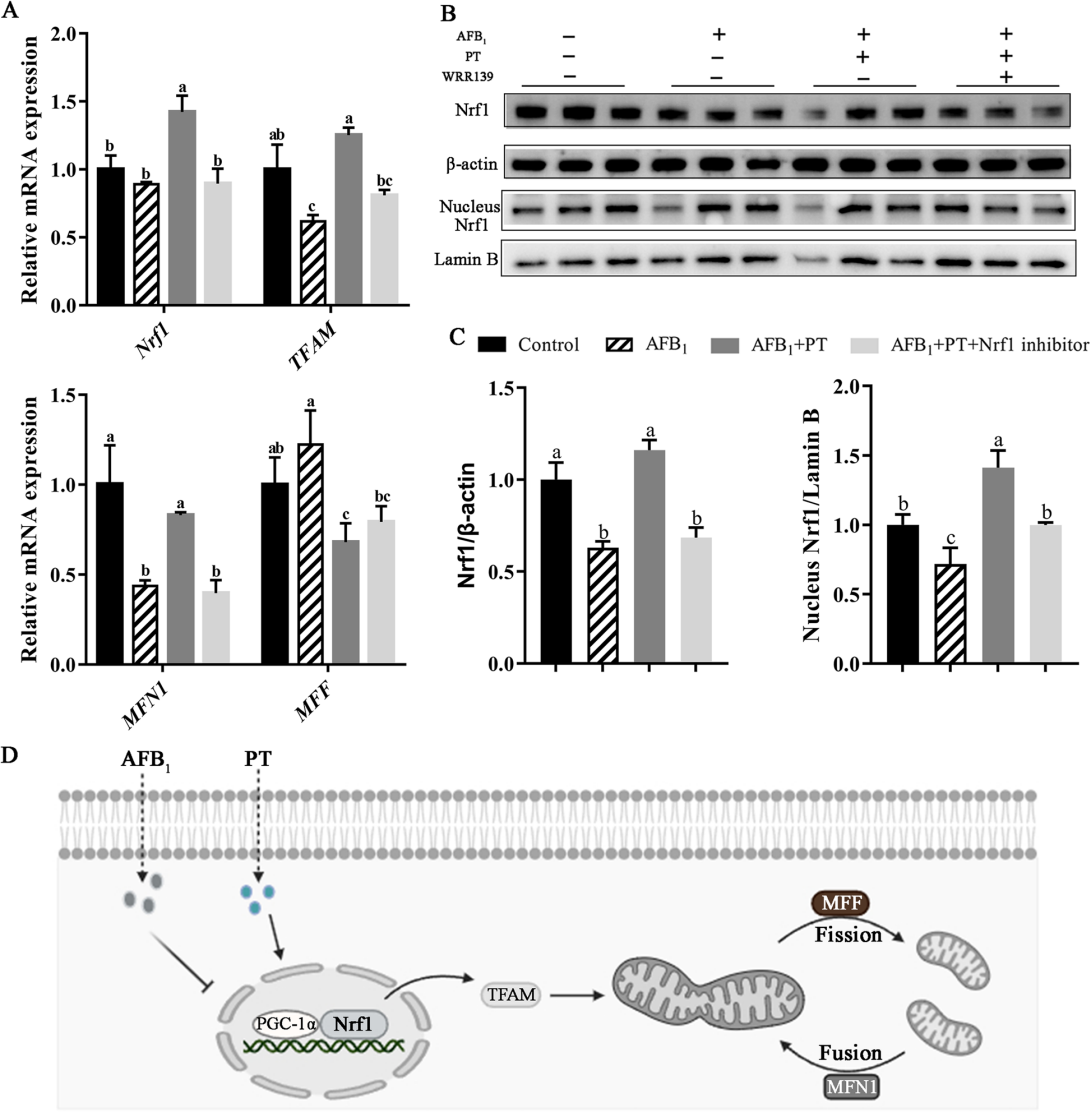
Effect of phlorotannin on AFB_1_-induced mitochondrial damage of LMH cells via Nrf1 pathway. **A** Relative mRNA levels of Nrf1 pathway-related gene in AFB_1_-induced LMH cells. **B** Western blot analysis of total and nuclear Nrf1 in AFB_1_-induced LMH cells. Lamin B and β-actin served as loading control. **C** Quantification of protein expression total and nuclear Nrf1. **D** Schematic diagram of mitochondrial function mediated by AFB_1_ and PT on Nrf1. AFB_1_, Aflatoxin B_1_; PT, Phlorotannin; Control, 10% complete medium + 0.025% DMSO; AFB_1_, 10% complete medium + 0.1 μg/mL AFB_1_; AFB_1_ + PT, 10% complete medium + 0.1 μg/mL AFB_1_ + 1 μg/mL PT; AFB_1_ + PT + Nrf1 inhibitor, 10% complete medium + 0.1 μg/mL AFB_1_ + 1 μg/mL PT + 10 μmol/L WRR139 (Nrf1 inhibitor). – for not adding, + for adding. Data are shown as mean ± SEM (*n* = 6). ^a–c^Different superscript letters indicate significant differences (*P* < 0.05)

The mRNA levels of *TFAM* and *MFN1* were decreased in the presence of AFB_1_ compared with the control (*P* < 0.05). When pretreated with 1 μg/mL PT, the mRNA levels of *Nrf1, TFAM,* and *MFN1* were dramatically increased compared with the AFB_1_ group, but Nrf1 inhibitor (WRR139) treatment inhibited the effect of PT (*P* < 0.05; Fig. 7A). By western blotting, compared with the control group, the total and nuclear Nrf1 protein expression levels of LMH cells in AFB_1_ group were down-regulated (*P* < 0.05). The total and nuclear Nrf1 protein expression levels were upregulated in PT treatment compared with the AFB_1_ group (*P* < 0.05); such an elevation was reduced when exposed to an Nrf1 inhibitor (WRR139) (*P* < 0.05;Fig. 7B and C).

## Discussion

Contamination of AFB_1_ is a challenge to the livestock feed industry. Poultry as one of the most sensitive animals to AFB_1_, particularly the chicks, their liver detoxification ability is relatively weak, making them susceptible to AFB_1_ [40]. The investigation report of animal feed pollution in China showed that the detection rate of AFB_1_ in poultry diets was 99.6%, and the maximal concentration was 54 μg/kg [4]. The Chinese feed hygiene standards set the maximum limit of AFB_1_ in broiler feed at 10–20 μg/kg [41]. It has been reported that when exposed to 50 μg/kg AFB_1_, the ADG of broilers decrease by 5%–10%, and when exposed to 100 μg/kg AFB_1_, the ADG decrease by 10%–20% in broilers [42]. Gao et al. [43] found that broiler chicks exposed to 0.1 mg/kg of AFB_1_ for 21 d had reduced growth performance and liver damage. Ma et al. [44] found that the FCR of AA broiler with 0.1 mg/kg AFB_1_ in the basal diet was higher than that of the control group, and reached 1.87%. In our study, after feeding 0.1 mg/kg of AFB_1_ for 21 d, the ADFI and FCR of broilers increased, and the liver index decreased. It can be seen that feeding broiler chickens 0.1 mg/kg AFB_1_ has a negative effect on their growth performance and liver. However, currently, there is limited research on the use of PT in broilers, but other polyphenols have been proven to improve the growth performance of poultry [45]. Jiang et al. [46] showed that curcumin can reduce the adverse effects of AFB_1_ and promote the growth and development of ducklings. Similarly, Yang et al. [47] found that resveratrol could improve the growth performance by increasing the ADG and final weight of ducklings. We found that supplementation of 400, 600, and 800 mg/kg PT decreased the ADFI, and 400 mg/kg PT decreased the FCR of broilers. In addition, supplementation of 600 mg/kg PT can significantly increase the liver index of broilers. To sum up, the results showed that PT can alleviate the growth performance decline of broilers caused by AFB_1_ to some extent. It can be attributed to PT improving the liver function damage of broilers induced by AFB_1_ and promoting the metabolism of nutrients and excretion of non-nutrients.

As the main target organ of AFB_1_, AFB_1_ can cause severe damage to the liver and hepatocytes [1]. The damage to the liver can lead to abnormal metabolism of nutrients, which can be reflected by blood biochemical indexes. However, the activities of AST, ALT, and ALP in serum are commonly used to detect whether the liver function is normal [48]. AST and ALT are synthesized by liver cells [49]. Exposure of broilers to AFB_1_ causes serious hepatic injury, which leads to the release of aminotransferase from liver cells into the blood when the permeability of liver cells increases, thus improving the serum activities of ALT and AST, which is consistent with the results of this study [50]. A previous study has shown that resveratrol can improve the oxidative stress of the liver induced by AFB_1_, such as reducing the activities of ALT, AST, and ALP in the liver of broilers [51]. Supplementation of 200, 400, 600, and 800 mg/kg PT in this study increased the TP content. Supplementation of 200, 600, and 800 mg/kg PT decreased the activity of AST. It is worth noting that supplementation of 600 mg/kg PT increased the ALB content and decreased the ALT activity. Therefore, based on some indexes, we considered 600 mg/kg PT as the best dose to alleviate the liver damage induced by AFB_1_ in broilers. A previous study reported that AFB_1_ can cause histopathological changes in the liver, such as necrosis, degeneration, and inflammation [52]. In addition, AFB_1_ can induce apoptosis and increase the percentage of apoptosis, thus causing liver damage. Li et al. [45] found that broilers fed AFB_1_-contaminated diets showed inflammatory cell infiltration, hepatic lobule structure, and hepatic cord disorder in the liver, and these pathological changes were improved after curcumin supplementation. Similar to the above research results, with supplementation of 600 mg/kg PT, the overall pathological changes of the liver were alleviated, the inflammatory infiltration of hepatocytes was significantly reduced, the vacuoles in the cytoplasm disappeared, and TUNEL staining revealed a reduction in AFB_1_-induced hepatocyte apoptosis. Also, in vitro assays found that the survival rate of hepatocytes significantly increased. It suggests that PT could protect the liver from the damage caused by AFB_1_ and maintain the normal structure and function of hepatocytes.

It is well known that the liver is the main organ for the accumulation and metabolism of AFB_1_, mainly mediated by phase I and phase II detoxification enzymes, and phase I and II detoxification are two different types of metabolic processes [53]. Phase I reaction is mainly oxidation, reduction, and hydrolysis, which is usually a coupling reaction as a chemical construction of phase II, and Nrf2 is not only a key transcription factor to regulate oxidative stress response but also a key transcription factor to regulate phase II detoxification enzymes [53]. For the hepatotoxicity induced by AFB_1_, Nrf2 can enhance antioxidant capacity by promoting the expression of phase II etoxification enzymes. GSTs, as the key of phase II detoxification enzyme, which isozymes are divided into classes of Alpha (GSTA), Pi (GSTP), Theta (GSTT), Omega (GSTO) and so on [54]. Studies have shown that the expression of GSTA3, GSTO1, and GSTT1 play an important role in regulating redox balance and metabolizing exogenous toxins [55, 56]. It is believed that the ability of AFBO to undergo conjugate binding reaction in vivo depends on the content, type, and activity of GST, which catalyzes the coupling of AFB_1_ with glutathione (GSH) to produce a non-toxic product (AFBO-GSH), which are excreted in urine [52]. In this study, AFB_1_ resulted in the decreased T-AOC level, activities of GST, GSH-Px, and HO-1, and relative expression of *MafF*,* MafK*, and *Nrf2* in broilers liver, and PT supplementation upregulated the activities of antioxidant enzymes and phase II detoxification enzymes and the expression of Nrf2 signaling pathway-related genes. Zhang et al. [57] reported that curcumin alleviated AFB_1_-induced liver damage in broilers by promoting the expression of Nrf2 pathway-related genes, which is consistent with our findings.

We conducted in vitro trials to verify our results and to determine whether PT can alleviate AFB_1_-induced liver injury through the Nrf2 signaling pathway. The results showed that by adding 1 μg/mL PT to LMH cells exposed to AFB_1_, the relative expression of Nrf2-related genes and proteins was significantly upregulated after supplementing PT. The Nrf2 signaling pathway was affected after supplementation of Nrf2 inhibitor (ML385), and the nucleation of p-Nrf2 protein was reduced. Luo et al. [33] showed that nano-ions based on phlorotannin effectively attenuated carbon tetrachloride (CCl_4_)-induced liver damage, oxidative stress, and inflammation in mice liver by modulating the Nrf2/HO-1 signaling pathway. Park et al. [59] found that phlorotannin increased the phosphorylation of Nrf2 in myoblasts and upregulated the expression of *HO-1* and its enzymatic activity, which was similar in this study. Our results confirm that PT inhibited AFB_1_-induced liver damage in broilers by activating the phase II detoxification enzyme pathway mediated by Nrf2 signaling pathway. The antioxidant system of the body is regulated by antioxidant enzymes and the glutathione redox system [60]. Antioxidant enzymes are an important factor in maintaining redox balance, and the elimination of free radicals in tissues depends on a variety of antioxidant enzymes, including SOD, CAT, and GSH-Px [61]. Studies have confirmed that the damage of AFB_1_ to the liver is mainly due to the destruction of the antioxidant system of the liver, which leads to liver damage and dysfunction [12]. In this study, AFB_1_ decreased the level of T-AOC, decreased the activities of CAT, T-SOD, GST, GSH-Px, and HO-1, and increased the content of MDA in the broiler’s liver, which was consistent with a previous study [62]. Wang et al. [63] showed that the addition of catechin effectively reduced the level of MDA and increased the level of T-AOC and the activity of CAT enzyme. Jin et al. [64] showed that adding curcumin to the diet could restore the activity of antioxidant enzymes in duck liver induced by AFB_1_ to the normal level. Consistent with the above research, supplementation of 600 mg/kg PT in this study effectively alleviated the oxidative stress induced by AFB_1_. Therefore, supplementation of PT can regulate the expression of Nrf2 to alleviate the oxidative stress induced by AFB_1_ and promote the expression of phase II detoxification enzyme to play a detoxification role.

Mitochondria are crucial for energy metabolism in cells and are often called the “energy pool” of the cell [65]. As a central metabolic organ, the liver contains a large number of mitochondria [66], and as such, mitochondria are essential for metabolic homeostasis in the liver, and their dysfunction is also a major cause of liver disease [67]. However, the normal structure of mitochondria is the basis of its various functions, and the damage to mitochondrial structure relates to its functions [24]. AFB_1_, as an important inducer of mitochondrial antioxidant dysfunction, can induce a large number of ROS, trigger oxidative stress, and further induce DNA and mitochondrial damage and inflammatory reaction [22]. When ROS is produced excessively, it disturbs the steady state of cells, leads to excessive mitochondria fusion and division, causes mitochondrial dysfunction, and leads to cell damage and even death [68]. In addition, AFB_1_ can also decrease the level of mitochondrial membrane potential and induce the increase of mitochondrial permeability by uncoupling mitochondrial oxidative phosphorylation. The ultrastructure of hepatic mitochondria was observed by transmission electron microscope. The structure of mitochondria was damaged by AFB_1_, and most of the mitochondrial bilayer membrane was fuzzy and cristae were disordered, and the double-layer membrane structure was restored. These outcomes are similar to those of a previous study, which reported that when curcumin was added to the feed of broilers exposed to AFB_1_, mitochondrion was clearly visible and evenly distributed, and its structure returned to normal levels [69]. Studies have shown that AFB_1_ produces excess ROS, induces mitochondrial DNA damage and autophagy, reduces its membrane potential, and induces protein and lipid oxidation [1, 69, 70]. The liver is a highly oxygen-consuming organ, and its rich mitochondria account for about 18% of the cell volume. Therefore, the damaged mitochondria can be used as a marker of liver cell damage, and severe mitochondrial damage affects vital functions such as metabolism and detoxification in the liver [71]. There are evidence that AFB_1_ reduces mitochondrial quality and interferes with mitochondrial biogenesis [24, 72]. Li et al. [2] found that the structure of mitochondria in the liver of broilers exposed to AFB_1_ was obviously damaged, and the mitochondria showed severe swelling or vacuolization and disappearance of the mitochondrial crest, which is consistent with our findings. Mitochondrial biosynthesis is very important for repairing mitochondrial structure and maintaining its function, and this process is regulated by the key transcription activator PGC-1α [73]. Activation of PGC-1α can promote mitochondrial biosynthesis by activating the Nrf1/TFAM pathway [74]. However, as a key regulator of mitochondrial biosynthesis and function, Nrf1 is released from the endoplasmic reticulum to the cytoplasm by a series of complex reversal, modification, and processing processes, and finally into the nucleus to regulate the expression of downstream genes, such as mitochondrial transcription factor a (TFAM), Cytochrome c [75]. A series of studies have shown that PT and other polyphenols play key roles in ameliorating hepatotoxicity, interacting with Nrf1 signaling pathways to inhibit the damage of hepatic mitochondria [76, 77]. Huang et al. [78] found that the mitochondrial structure of mice exposed to AFB_1_ was damaged, which showed that the mRNA expression levels of *PGC-1α*,* Nrf1*,* TFAM*,* DRP1*,* FIS1*,* MFN1* and *OPA1* decreased. Our study proved that the characteristics of mitochondrial dysfunction by PT in AFB_1_-induced hepatotoxicity were related to the upregulated Nrf1/TFAM pathway. That is, AFB_1_ downregulated the Nrf1/TFAM pathway, while PT upregulated it; after Nrf1 was silenced, the role of PT was partially inhibited. Therefore, the present study suggests that PT can alleviate AFB_1_-induced mitochondrial damage in hepatocytes by activating Nrf1 signaling pathway, thereby further alleviating liver damage in broilers.

## Conclusion

Supplementation of 600 mg/kg phlorotannin alleviated AFB_1_-induced liver damage in broilers, and 1 μg/mL phlorotannin has a hepatoprotective role for hepatocytes exposed to AFB_1_ in vitro. This effect is mainly through activating the Nrf2 signal pathway, inhibiting the activity of phase I detoxification enzymes and increasing the activity of phase II detoxification enzymes, thus promoting the excretion of AFB_1_ in the liver. Also, phlorotannin could upregulate the Nrf1 signaling pathway to alleviate AFB_1_-induced mitochondrial damage, thereby further alleviating liver injury. These findings provide novel insights into studying phlorotannin as a new type of biological detoxification substance to protect against liver damage caused by AFB_1_.

## Supplementary Information

Additional file 1: Table S1 Primer sequences for hepatic gene expression. Table S2 Antibody information.

## Data Availability

The basic data of this paper will be shared by the corresponding authors under reasonable requirements.

## Abbreviations

ADFI: Average daily feed intake
ADG: Average daily gain
AFB_1_: Aflatoxin B_1_
AFBO: AFB1-exo-8,9-epoxide
ALB: Albumin
ALT: Alanine aminotransferase
AST: Aspartate aminotransferase
CAT: Catalase
CCK- 8: Cell counting Kit-8
CYP1A1: Cytochrome P450 family 1 subfamily A member 1
CYP1A2: Cytochrome P450 family 1 subfamily A member 2
CYP2A6: Cytochrome P450 family 2 subfamily A member 6
CYP3A4: Cytochrome P450 family 3 subfamily A member 4
FBS: Fetal bovine serum
FCR: Feed conversion rate
GCLM: Glutamate-cysteine ligase
GSH: Glutathione
GSH-PX: Glutathione peroxidase
GSH-ST: Glutathione S-transferase
H&E: Hematoxylin and eosin
HO-1: Heme oxygenase 1
LMH: Leghorn Male Hepatoma
MDA: Malondialdehyde
MFN1: Mitofusin 1
MFN2: Mitofusin 2
Nrf1: Nuclear respiratory factor 1
Nrf2: Nuclear factor erythroid 2-related factor 2
NQO1: NAD(P)H:quinone dehydrogenase 1
OPA1: Optic atrophy 1
PGC-1α: Peroxisome proliferators-activated receptor γ coactivator l alpha
PT: Phlorotannin
ROS: Reactive oxygen species
T-AOC: Total antioxidant capacity
TFAM: Mitochondrial transcription factor A
TUNEL: Terminal deoxynucleotidyl transferase-mediated dUTP nick-end labeling
TP: Total protein
T-SOD: Total superoxide dismutase

## References

[1] Wu G, San J, Pang H, Du Y, Li W, Zhou X, et al. Taurine attenuates AFB_1_-induced liver injury by alleviating oxidative stress and regulating mitochondria-mediated apoptosis. Toxicon. 2022;215:17–27. 35688267 10.1016/j.toxicon.2022.06.003

[2] Li S, Liu R, Xia S, Wei G, Ishfaq M, Zhang Y, et al. Protective role of curcumin on aflatoxin B_1_-induced TLR4/RIPK pathway mediated-necroptosis and inflammation in chicken liver. Ecotoxicol Environ Saf. 2022;233:113319. 35189522 10.1016/j.ecoenv.2022.113319

[3] Rodrigues I, Naehrer K. A three-year survey on the worldwide occurrence of mycotoxins in feedstuffs and feed. Toxins. 2012;4:663–75. 23105974 10.3390/toxins4090663 PMC3475222

[4] Zhao L, Zhang L, Xu Z, Liu X, Chen L, Dai J, et al. Occurrence of Aflatoxin B_1_, deoxynivalenol and zearalenone in feeds in China during 2018–2020. J Anim Sci Biotechnol. 2021;12:74. 34243805 10.1186/s40104-021-00603-0 PMC8272344

[5] Pereira CS, Cunha SC, Fernandes JO. Prevalent mycotoxins in animal feed: Occurrence and analytical methods. Toxins. 2019;11:290. 31121952 10.3390/toxins11050290 PMC6563184

[6] Mikela V, Andreana P, Nikolaos S, Alexander G. Ochratoxin A in slaughtered pigs and pork products. Toxins. 2022;14:67. 35202095 10.3390/toxins14020067 PMC8876995

[7] Chen X, Ishfaq M, Wang J. Effects of *Lactobacillus salivarius* supplementation on the growth performance, liver function, meat quality, immune responses and* Salmonella* Pullorum infection resistance of broilers challenged with Aflatoxin B_1_. Poult Sci. 2022;101(3):101651. 10.1016/j.psj.2021.101651 PMC8753273 34999537

[8] Oloruntola OD, Ayodele SO, Oloruntola DA, Olarotimi OJ, Falowo AB, Akinduro VO, et al. Dietary supplementation of Capsicum powder affects the growth immun. Toxicon. 2024;240:107640. 38325757 10.1016/j.toxicon.2024.107640

[9] Frangiamone M, Lázaro Á, Cimbalo A, Font G, Manyes L. In vitro and in vivo assessment of AFB1 and OTA toxic effects and the beneficial role of bioactive compounds. A systematic review Food Chem. 2024;447:138909. 38489879 10.1016/j.foodchem.2024.138909

[10] Rushing BR, Selim MI. Aflatoxin B1: A review on metabolism, toxicity, occurrence in food, occupational exposure, and detoxification methods. Food Chem Toxicol. 2018;124:81–100. 30468841 10.1016/j.fct.2018.11.047

[11] Frijhoff J, Winyard P, Zarkovic N, Davies S, Stocker R, Cheng D, et al. Clinical relevance of biomarkers of oxidative stress. Antioxid Redox Signal. 2015;23:1144–70. 26415143 10.1089/ars.2015.6317 PMC4657513

[12] Xu Q, Shi W, Lv P, Meng W, Mao G, Gong C, et al. Critical role of caveolin-1 in aflatoxin B1-induced hepatotoxicity via the regulation of oxidation and autophagy. Cell Death Dis. 2020;11:6. 31919341 10.1038/s41419-019-2197-6 PMC6952418

[13] Guerre P, Pineau T, Costet P, Burgat V, Galtier P. Effects of AFB1 on CYP 1A1, 1A2 and 3A6 mRNA and P450 expression in primary culture of rabbit hepatocytes. Toxicol Lett. 2000;111:243–51. 10643869 10.1016/s0378-4274(99)00181-2

[14] Vlastimil D, Qinghua W, Kamil K. Metabolism of aflatoxins: key enzymes and interindividual as well as interspecies differences. Arch Toxicol. 2014;88:1635–44. 25027283 10.1007/s00204-014-1312-9

[15] Qiao B, He Y, Gao X, Liu H, Gan R, Qian H, et al. Curcumin attenuates AFB1-induced duck liver injury by inhibiting oxidative stress and lysosomal damage. Food Chem Toxicol. 2023;172:107177. 10.1016/j.fct.2022.113593 36596445

[16] Ishfaq M, He W, Xiaoqi S, Wang X, Han M, Lu Z, et al. Dual Role of dietary curcumin through attenuating AFB1-induced oxidative stress and liver injury via modulating liver phase-I and phase-II enzymes involved in AFB1 bioactivation and detoxification. Front Pharmacol. 2018;9:554. 29887802 10.3389/fphar.2018.00554 PMC5981209

[17] Jin X, Li Q, Sun J, Zhang M, Xiang Y. Porcine β-defensin-2 alleviates AFB1-induced intestinal mucosal injury by inhibiting oxidative stress and apoptosis. Ecotoxicol Environ Saf. 2023;262:115161. 37356398 10.1016/j.ecoenv.2023.115161

[18] Wang P, Wang Y, Feng T, Yan Z, Zhu D, Lin H, et al. Hedyotis diffusa alleviate aflatoxin B1-induced liver injury in ducks by mediating Nrf2 signaling pathway. Ecotoxicol Environ Saf. 2023;249:114339. 36508825 10.1016/j.ecoenv.2022.114339

[19] Liu Y, Wang W. Aflatoxin B1 impairs mitochondrial functions, activates ROS generation, induces apoptosis and involves Nrf2 signal pathway in primary broiler hepatocytes. Anim Sci J. 2016;87:1490–500. 26997555 10.1111/asj.12550

[20] Mary VS, Theumer MG, Arias SL, Rubinstein HR. Reactive oxygen species sources and biomolecular oxidative damage induced by aflatoxin B1 and fumonisin B1 in rat spleen mononuclear cells. Toxicology. 2012;302:299–307. 22981896 10.1016/j.tox.2012.08.012

[21] Guo Y, Balasubramanian B, Zhao Z, Liu W. Marine algal polysaccharides alleviate aflatoxin B1-induced bursa of Fabricius injury by regulating redox and apoptotic signaling pathway in broilers. Poult Sci. 2021;100:844–57. 33518138 10.1016/j.psj.2020.10.050 PMC7858151

[22] Zhang L, Shi S, Liu Y, Cui Y, Zhu Y, Bao Y, et al. Aflatoxin B1 triggers apoptosis in rabbit hepatocytes via mediating oxidative stress and switching on the mitochondrial apoptosis pathway. Ecotoxicol Environ Saf. 2023;264:115478. 37716070 10.1016/j.ecoenv.2023.115478

[23] Chen P, Ding W, Xu B, Rehman M, Liu K, He Y, et al. Aflatoxin B1 as a complicit in intestinal damage caused by *Eimeria ovinoidalis* in lambs: Novel insights to reveal parasite-gut battle. Sci Total Environ. 2024;947:174539. 38977103 10.1016/j.scitotenv.2024.174539

[24] Xu F, Li Y, Cao Z, Zhang J, Huang W. AFB1-induced mice liver injury involves mitochondrial dysfunction mediated by mitochondrial biogenesis inhibition. Ecotoxicol Environ Saf. 2021;216:112213. 33838459 10.1016/j.ecoenv.2021.112213

[25] Liu W, Yang Y, Pushparaj K, Balasubramanian B. Evaluation of hepatic detoxification effects of *Enteromorpha prolifera* polysaccharides against aflatoxin B_1_ in broiler chickens. Antioxidants. 2022;11(9):1757. 36139831 10.3390/antiox11091757 PMC9495745

[26] Nabi F, Tao W, Li Z, Lu Q, Xie J, Sahito B, et al. *Penthorum chinense* Prush extract alleviates aflatoxin B_1_-induced toxicity, oxidative stress and apoptosis via mediating Nrf2 signaling pathway in the Bursa of Fabricius of broilers. Comp Biochem Physiol C Toxicol Pharmacol. 2024;275:109779. 37871871 10.1016/j.cbpc.2023.109779

[27] Dong W, Liu M, Liu B, Xiao Y, Liu X, Yang M, et al. Isolation of *Bacillus licheniformis* and its protective effect on liver oxidative stress and apoptosis induced by aflatoxin B_1_. Poult Sci. 2024;103:104079. 39098297 10.1016/j.psj.2024.104079 PMC11345652

[28] Meng W, Sun H, Mu T, Marco G. Extraction, purification, chemical characterization and antioxidant properties in vitro of polyphenols from the brown macroalga *Ascophyllum nodosum*. Algal Res. 2023;70:102989.

[29] Leonel P, João C. Therapeutic potential of polyphenols and other micronutrients of marine origin. Mar Drugs. 2023;21(6):323. 37367648 10.3390/md21060323 PMC10303569

[30] Cheng K, Niu J, Zhang J, Qiao Y, Dong G, Guo R, et al. Hepatoprotective effects of chlorogenic acid on mice exposed to aflatoxin B_1_: Modulation of oxidative stress and inflammation. Toxicon. 2023;231:107177. 37276986 10.1016/j.toxicon.2023.107177

[31] Maheswari V, Saravana B. Phlorotannin and its derivatives, a potential antiviral molecule from brown seaweeds, an overview. Russ J Mar Biol. 2022;48:309–24. 36405241 10.1134/S1063074022050169 PMC9640822

[32] Akinyemi F, Adewole D. Effects of brown seaweed products on growth performance, plasma biochemistry, immune response, and antioxidant capacity of broiler chickens challenged with heat stress. Poult Sci. 2022;101:102215. 36288626 10.1016/j.psj.2022.102215 PMC9593180

[33] Luo F, Zhu B, Wu D, Xu Y, Tao C, Lin Y, et al. Construction of phlorotannin-based nanoparticles for alleviating acute liver injury. ACS Appl Mater Interfaces. 2023;15(40):47338–49. 37751516 10.1021/acsami.3c05407

[34] Kang M, Ahn G, Yang X, Kim K, Kang S, Lee S, et al. Hepatoprotective effects of dieckol-rich phlorotannins from Ecklonia cava, a brown seaweed, against ethanol induced liver damage in BALB/c mice. Food Chem Toxicol. 2012;50:1986–91. 22504843 10.1016/j.fct.2012.03.078

[35] Guo Y. Preparation of nano-Se-Enteromorpha polysaccharide and its protective effect on liver injury induced by AFB1 in broilers. Master thesis. Guangdong Ocean University, Animal Science Department; 2022.

[36] Cheng W, Zuo R, Chang J, Yin Q, Wang P, Dang X. Effect of probiotics and aflatoxin B1 degrading enzyme on growth performance of broilers and its mechanisms. Chin J Anim Nutr. 2014;26:1608–15.

[37] Jobling M. National Research Council (NRC): Nutrient requirements of fish and shrimp. Aquac Int. 2012;20:601–2.

[38] Adhikari M, Negi B, Kaushik N, Adhikari A, Al-Khedhairy A, Kaushik N, et al. T-2 mycotoxin: toxicological effects and decontamination strategies. Oncotarget. 2017;8:33933–52. 28430618 10.18632/oncotarget.15422 PMC5464924

[39] Li S, Han M, Zhang Y, Ishfaq M, Liu R, Wei G, et al. Effect of curcumin as feed supplement on immune response and pathological changes of broilers exposed to aflatoxin B1. Biomolecules. 2022;12(9):1188. 10.3390/biom12091188 PMC9496629 36139027

[40] Wang H, Muhammad I, Li W, Sun X, Cheng P, Zhang X. Sensitivity of Arbor Acres broilers and chemoprevention of aflatoxin B1-induced liver injury by curcumin, a natural potent inducer of phase-II enzymes and Nrf2. Environ Toxicol Pharmacol. 2018;59:94–104. 29550706 10.1016/j.etap.2018.03.003

[41] Ma R, Zhang L, Liu M, Su Y, Xie W, Zhang N, et al. Individual and combined occurrence of mycotoxins in feed ingredients and complete feeds in China. Toxins. 2018;10(3):113. 10.3390/toxins10030113 PMC5869401 29518909

[42] Fouad AM, Ruan D, El-Senousey HK, Chen W, Jiang S, Zheng C. Harmful effects and control strategies of aflatoxin B1 produced by *Aspergillus flavus* and *Aspergillus parasiticus* strains on poultry: Review. Toxins. 2019;11(3):176. 10.3390/toxins11030176 PMC6468546 30909549

[43] Gao S, Zhang L, Zhu D, Huang J, Yang J, Jiang J, et al. Effects of glucose oxidase and bacillus subtilis on growth performance and serum biochemical indices of broilers exposed to aflatoxin B1 and endotoxin. Anim Feed Sci Technol. 2022;286: 115186.

[44] Ma H, Chen Q, Yang H, Wan X. Effects of lycopene on the growth performance, meat quality, and antioxidant capacity of broiler chickens challenged with aflatoxin B1. J Food Sci. 2024;89:96–103. 37983886 10.1111/1750-3841.16848

[45] Li S, Liu R, Wei G, Guo G, Yu H, Zhang Y, et al. Curcumin protects against Aflatoxin B_1_-induced liver injury in broilers via the modulation of long non-coding RNA expression. Ecotoxicol Environ Saf. 2021;208:111725. 33396056 10.1016/j.ecoenv.2020.111725

[46] Jiang X, Liu H, You Y, Zhong G, Ruan Z, Liao J, et al. Multi-omics reveals the protective effects of curcumin against AFB1-induced oxidative stress and inflammatory damage in duckling intestines. Comp Biochem Physiol C Toxicol Pharmacol. 2024;276:109815. 38061615 10.1016/j.cbpc.2023.109815

[47] Yang H, Wang Y, Liu M, Liu X, Jiao Y, Shan S, et al. Effects of dietary resveratrol supplementation on growth performance and anti-Inflammatory ability in ducks (*Anas platyrhynchos*) through the Nrf2/HO-1 and TLR4/NF-κB signaling pathways. Animals. 2021;11:3588. 10.3390/ani11123588 PMC8698092 34944363

[48] Lin R, Wu P, Wu Y, Huang L, Lin B, Huang L. Effects of compound *Anoectochilus roxburghii* (Wall.) Lindl.l oral liquid on relative metabolic enzymes and various biochemical indices in Wistar rats with isoniazid-induced liver injury. J Pharm Biomed Anal. 2024;248:116249. 10.1016/j.jpba.2024.116249 38936169

[49] Yokoyama H, Masuyama T, Tanaka Y, Shimazaki T, Yasui Y, Abe T, et al. Diacylglycerol O-acyltransferase 1 inhibitor increases plasma alanine aminotransferase and aspartate aminotransferase activities via a shedding of the intestinal villi and an increase in intestinal permeability in rats. Toxicol Lett. 2024;400:16–23. 39096942 10.1016/j.toxlet.2024.08.004

[50] Alharthi A, Al Sulaiman A, Aljumaah R, Alabdullatif A, Elolimy A, Alqhtani A, et al. Protective effect of date pits on growth performance, carcass traits, blood indices, intestinal morphology, nutrient digestibility, and hepatic aflatoxin residues of aflatoxin B1-exposed broilers. Agriculture. 2022;12:476.

[51] Sridhar M, Suganthi RU, Thammiaha V. Effect of dietary resveratrol in ameliorating aflatoxin B1-induced changes in broiler birds. J Anim Physiol Anim Nutr. 2015;99:1094–104. 10.1111/jpn.12260 25319220

[52] Wang X, Li W, Wang X, Han M, Muhammad I, Zhang X, et al. Water-soluble substances of wheat: a potential preventer of aflatoxin B1-induced liver damage in broilers. Poult Sci. 2019;98:136–49. 30107611 10.3382/ps/pey358

[53] Sarker M, Wan X, Yang H, Wang Z. AflatoxinB1 (AFB1) and its toxic effect on the broilers intestine: A review. Vet Med Sci. 2023;9(4):1646–55. 37401533 10.1002/vms3.1169 PMC10357249

[54] Mannervik B, Board P, Hayes J, Listowsky I, Pearson W. Nomenclature for mammalian soluble glutathione transferases. Methods Enzymol. 2005;401:1–8. 16399376 10.1016/S0076-6879(05)01001-3

[55] Board P, Coggan M, Chelvanayagam G, Easteal S, Jermiin L, Schulte G, et al. Identification, characterization, and crystal structure of the omega class glutathione transferases. J Biol Chem. 2000;275:24798–806. 10.1074/jbc.M001706200 10783391

[56] Li J, Guo C, Liu Y, Han B, Lv Z, Jiang H, et al. Chronic arsenic exposure-provoked biotoxicity involved in liver-microbiota-gut axis disruption in chickens based on multi-omics technologies. J Adv Res. 2025;67:373–86. 38237767 10.1016/j.jare.2024.01.019 PMC11725159

[57] Zhang J, Sun X, Chai X, Jiao Y, Sun J, Wang S, et al. Curcumin mitigates oxidative damage in broiler liver and ileum caused by aflatoxin B1-contaminated feed through Nrf2 signaling pathway. Animals. 2024;14(3):409. 38338051 10.3390/ani14030409 PMC10854683

[58] Luo F, Zhu B, Wu D, Xu Y, Chen T, Li Y, et al. Construction of phlorotannin-based nanoparticles for alleviating acute liver injury. ACS Appl Mater Interfaces. 2023;15:47338–49. 37751516 10.1021/acsami.3c05407

[59] Park C, Cha H, Hwangbo H, Yeong J, Kim D, Kim M, et al. Phloroglucinol inhibits oxidative-stress-induced cytotoxicity in C2C12 murine myoblasts through Nrf-2-mediated activation of HO-1. Int J Mol Sci. 2023;24:4637. 36902068 10.3390/ijms24054637 PMC10003575

[60] Lin L, Fu P, Chen N, Gao N, Cao Q, Yue K, et al. Total flavonoids of *Rhizoma Drynariae* protect hepatocytes against aflatoxin B1-induced oxidative stress and apoptosis in broiler chickens. Ecotoxicol Environ Saf. 2022;230:113148. 34995912 10.1016/j.ecoenv.2021.113148

[61] Hong H, Xi B, Kexing X, Cheng Z, Liang C. Effect of phloretin on growth performance, serum biochemical parameters and antioxidant profile in heat-stressed broilers. Poult Sci. 2021;100:101217. 34161850 10.1016/j.psj.2021.101217 PMC8237358

[62] Li W, Li W, Zhang C, Xu N, Fu C, Wang C, et al. Study on the mechanism of aflatoxin B1 degradation by Tetragenococcushalophilus. LWT. 2023;180.

[63] Wang Y, Wu J, Wang L, Yang P, Liu Z, Rajput S, et al. Epigallocatechin gallate and glutathione attenuate aflatoxin B1-induced acute liver injury in ducklings via mitochondria-mediated apoptosis and the Nrf2 signalling pathway. Toxins. 2022;14:876. 36548773 10.3390/toxins14120876 PMC9782748

[64] Jin S, Yang H, Wang Y, Pang Q, Jiao Y, Shan A, et al. Dietary curcumin alleviated aflatoxin B1-induced acute liver damage in ducks by regulating NLRP3–Caspase-1 signaling pathways. Foods. 2021;10:3086. 34945637 10.3390/foods10123086 PMC8701407

[65] Chen P, Yao L, Yuan M, Wang Z, Zhang Q, Jiang Y, et al. Mitochondrial dysfunction: A promising therapeutic target for liver diseases. Genes Dis. 2024;11:101115. 38299199 10.1016/j.gendis.2023.101115 PMC10828599

[66] Liu R, Yin C, Zhao P, Guo B, Ke W, Zheng X, et al. Nuclear respiratory factor 1 drives hepatocellular carcinoma progression by activating LPCAT1-ERK1/2-CREB axis. Biol Direct. 2023;18:67. 37875967 10.1186/s13062-023-00428-z PMC10594727

[67] Lee H, Lee T, Galloway C, Zhi W, Xiao W, de Mesy B, et al. The mitochondrial fusion protein OPA1 is dispensable in the liver and its absence induces mitohormesis to protect liver from drug-induced injury. Nat Commun. 2023;14:6721. 37872238 10.1038/s41467-023-42564-0 PMC10593833

[68] Wang Y, Wang X, Li Q. Aflatoxin B1 in poultry liver: Toxic mechanism. Toxicon. 2023;233:107262. 37619742 10.1016/j.toxicon.2023.107262

[69] Wang X, He Y, Tian J, Muhammad I, Liu M, Wu C, et al. Ferulic acid prevents aflatoxin B1-induced liver injury in rats via inhibiting cytochrome P450 enzyme, activating Nrf2/GST pathway and regulating mitochondrial pathway. Ecotoxicol Environ Saf. 2021;224:112624. 34416636 10.1016/j.ecoenv.2021.112624

[70] Gan Z, Guo Y, Zhao M, Ye Y, Liao Y, Liu B, et al. Excitatory amino acid transporter supports inflammatory macrophage responses. Sci Bull. 2024;69:2405–19. 10.1016/j.scib.2024.03.055 38614854

[71] Morio B, Panthu B, Bassot A, Rieusset J. Role of mitochondria in liver metabolic health and diseases. Cell Calcium. 2021;94:102336. 33387847 10.1016/j.ceca.2020.102336

[72] Chen X, Abdallah MF, Grootaert C, Van Nieuwerburgh F, Rajkovic A. New insights into the combined toxicity of aflatoxin B1 and fumonisin B1 in HepG2 cells using Seahorse respirometry analysis and RNA transcriptome sequencing. Environ Int. 2023;175:107945. 37126917 10.1016/j.envint.2023.107945

[73] Li L, Lu Z, Wang Y, Yang Y, Wang H, Ma H. Genistein alleviates chronic heat stress-induced lipid metabolism disorder and mitochondrial energetic dysfunction by activating the GPR30-AMPK-PGC-1α signaling pathways in the livers of broiler chickens. Poult Sci. 2023;103:103251. 37984004 10.1016/j.psj.2023.103251 PMC10694754

[74] Carlos C, Alexandra MV. Mitochondrial Biogenesis in Neurons: How and Where. Int J Mol Sci. 2021;22:13059. 34884861 10.3390/ijms222313059 PMC8657637

[75] Lou H, Yao J, Zhang Y, Wu X, Sun L, Wang Y, et al. Potential effect of acupuncture on mitochondrial biogenesis, energy metabolism and oxidation stress in MCAO rat via PGC-1α/NRF1/TFAM pathway. J Stroke Cerebrovasc Dis. 2024;33(11):107636. 38346661 10.1016/j.jstrokecerebrovasdis.2024.107636

[76] Yao X, Zhang J, Lu Y, Deng Y, Zhao R, Xiao S. Myricetin restores Aβ-Induced mitochondrial impairments in N2a-SW cells. ACS Chem Neurosci. 2022;13:454–63. 35114083 10.1021/acschemneuro.1c00591

[77] Wang H, Jiang T, Li W, Gao N, Zhang T. Resveratrol attenuates oxidative damage through activating mitophagy in an in vitro model of Alzheimer’s disease. Toxicol Lett. 2018;282:100–8. 29097221 10.1016/j.toxlet.2017.10.021

[78] Huang W, Cao Z, Yao Q, Ji Q, Zhang J, Li Y. Mitochondrial damage are involved in Aflatoxin B1-induced testicular damage and spermatogenesis disorder in mice. Sci Total Environ. 2020;701:135077. 31733399 10.1016/j.scitotenv.2019.135077

