# Supplementary material for: The ameliorative role of phlorotannin on aflatoxin B1-induced liver oxidative stress and mitochondrial injury is related to the activation of Nrf2 and Nrf1 signaling pathways in broilers
Source: J Anim Sci Biotechnol. 2025 May 22;16:75. doi: 10.1186/s40104-025-01210-z (PMC12096743; doi:10.1186/s40104-025-01210-z)
Supplement: Supplementary file 1 — Additional file 1: Table S1 Primer sequences for hepatic gene expression. Table S2 Antibody information. [file 40104_2025_1210_MOESM1_ESM.docx]

| **Table S1** Primer sequences for hepatic gene expression. | | |
| --- | --- | --- |
| Target Gene | Primer sequence 5’→3’ | Accession No. |
| *β-actin* | F: GTGATGGACTCTGGTGATGGTGTT  R: TCTCGGCTGTGGTGGTGAAG | NM_205518.1 |
| *CYP1A1* | F: CGGAGGCTGACAAGGTGCTGAT  R: AGGATGGTGGTGAGGAAGAGGAAG | NM_205147.1 |
| *CYP1A2* | F: CATCAACCTGGTGAATGACATCT  R: GCTCTGCCTGAATCTTCTTCTG | NM_205146.3 |
| *CYP2A6* | F: AGGATGGGAGACAGCAAAGG  R: ATCAAGGACACCAAGCTCAGA | KX687985.1 |
| *CYP3A4* | F: GCAAGAAGACAGTGGAGTTCAATG  R: AACGGCAGGAAGGTGTAGGG | NM_001329508.1 |
| *SOD1* | F: AGGGAGGAGTGGCAGAAGT  R: GCTAAACGAGGTCCAGCAT | NM_205064.1 |
| *SOD2* | F: TCCTGACCTGCCTTACGACTATGG  R: GCGACACCTGAGCTGTAACATCAC | NM_204211.1 |
| *GSTT1* | F: CATGCTAACATCCGGGCTAA  R: AAATTGCTTCAGGGAAGTGG | NM_205365.1 |
| *GSTA3* | F: GCGGCTGCTGGAGTTGAGTT  R: GTAGTTGAGGATGGCTCTGGTCTG | NM_001001777.1 |
| *GPX1* | F: CAAAGTGCTGCTGGTGGTCAAC  R: TTGGTGGCGTTCTCCTGGTG | NM_001277853.2 |
| *GPX3* | F: TGGCAGAGGAGTTCGGCAAC  R: CGTTCTTGACAGTGGCGATGTT | NM_001163232.2 |
| *GPX4* | F: GAATGTGCGCTCAGGCG  R: ACCGCGGTCTTTCCTCATTT | NM_204220.3 |
| *HO-1* | F: GCCTACACCCGCTATTTGG  R: TCTCAAGGGCATTCATTCG | NM_205344.1 |
| *GSTO1* | F: GGGCTGGTTCCTGTTCTG  R: TCTTCTGTAAGGCTCGCTCAT | NM_001277375.1 |
| *NQO1* | F: ACCATCTCTGACCTCTACGCCATA  R: GCCGCTTCAATCTTCTTCTGCTC | NM_001277619.1 |
| *Nrf2* | F: TGTGTGTGATTCAACCCGACT  R: TTAATGGAAGCCGCACCACT | NM_205117.1 |
| *Keap1* | F: ACTTCGCTGAGGTCTCCAAG  R: CAGTCGTACTGCACCCAGTT | NM_012289.4 |
| *MafF* | F: CGACGACGGACGCTGAAGAA  R: GTACTTGCCACGGAGAGTGTCAA | NM_204757.2 |

| **Continuation of Table S1** Primer sequences for hepatic gene expression. | | |
| --- | --- | --- |
| Target Gene | Primer sequence 5’→3’ | Accession No. |
| *MafK* | F: GCAGCAAGAGGTGGAGAAGC  R: ACGGCACGGAACTGGATGA | NM_204756.2 |
| *MafG* | F: ACGCTGAAGAACCGAGGCTAC  R: GTTCTGGCGAAGTTCTGGAGTG | NM_001079489.1 |
| *GCLC* | F: GACTGTGCTGGCAGAGAAGA  R: CTCCACCTGAGAACACGAGG | XM_046915268.1 |
| *CAT1* | F: CTCCTGGCTTACTCGTTGGT  R: CTCATTGTTATCTGTCTCCTCTGTT | NM_001145490 |
| *GCLM* | F: TGCTGAGTCACGGTGTCG  R: TGTTTTCTGAATGCAGTCCCG | NM_001007953 |
| *TFAM* | F: CACTCACTGACAGCAGCAGA  R: ACAGCAGCATAACTCAGGTT | NM_204100.2 |
| *SITR1* | F: AGTAGTAGCGAAAGCGGCTC  R: TCGTTCCCTGCAGCTTCATT | NM_001004767.2 |
| *Nrf1* | F: ACAAAAAGCCTCAACTCCTGC  R: GCCTCTTACGGATGGATGGG | XM_046907008.1 |
| *PGC-1α* | F: TCTCAGAAAGGGTCTCGTTGC  R: AGCACACTCGATGTCACTCC | NM_001006457.2 |
| *OPA1* | F: GCATTCAGAGCTACGGACCA  R: CGTGCTGCTGGATTTGTTGT | NM_001396172.1 |
| *MFN1* | F: TGGAGGAGCACTTGCTGAAG  R: GCTGCAGCTACGTTTATGGC | NM_001012931.3 |
| *MFN2* | F: TGAAGCGCAATGTCCCTGT  R: CATTGATGTACGCAGCCAGC | XM_040689233.2 |
| *DRP1* | F: TAGTATGTTGGGCCACTGCC  R: GCAGCTTATAGCAGGAATTCAGT | NM_001079722.2 |
| *Mff* | F: GGGTTTTCTGGCTTCTTTGCAT  R: GTGCTTCACCAAAGAGCGTC | XM_040679333.2 |

| **Table S2** Antibody information | | |
| --- | --- | --- |
| Protein antibodies | Dilution factor | Brand/No. |
| β-actin (I102) Polyclonal Antibody | 1:5000 | Bioworld/AP0060 |
| Rabbit Anti-Nrf2 Polyclonal Antibody | 1:1000 | Bioss/bs-1074R |
| Rabbit Anti-NRF1 Polyclonal Antibody | 1:1500 | Proteintech/12482-1-AP |
| Rabbit Anti-phospho-Nrf2(Ser40) Polyclonal Antibody | 1:1000 | Bioss/bs-2013R |
| Rabbit Anti-Lamin B1 Polyclonal Antibody | 1:2000 | Bioss/bs1840R |
| Goat Anti-Rabbit IgG (H+L) | 1:2000 | Youpin bio/YP848537-H |
